# Chromatin Fiber Invasion and Nucleosome Displacement by the Rap1 Transcription Factor

**DOI:** 10.1016/j.molcel.2019.10.025

**Published:** 2020-02-06

**Authors:** Maxime Mivelaz, Anne-Marinette Cao, Slawomir Kubik, Sevil Zencir, Ruud Hovius, Iuliia Boichenko, Anna Maria Stachowicz, Christoph F. Kurat, David Shore, Beat Fierz

**Affiliations:** 1 École Polytechnique Fédérale de Lausanne (EPFL), SB ISIC LCBM, Station 6, 1015 Lausanne, Switzerland; 2 Department of Molecular Biology and Institute of Genetics and Genomics of Geneva (iGE3), 1211 Geneva 4, Switzerland; 3 Molecular Biology Division, Biomedical Center, Faculty of Medicine, LMU Munich, 82152 Planegg-Martinsried, Germany

**Keywords:** pioneer transcription factor, Rap1, chromatin structure, chromatin dynamics, single-molecule fluorescence, FRET, RSC, chromatin remodeling

## Abstract

Pioneer transcription factors (pTFs) bind to target sites within compact chromatin, initiating chromatin remodeling and controlling the recruitment of downstream factors. The mechanisms by which pTFs overcome the chromatin barrier are not well understood. Here, we reveal, using single-molecule fluorescence, how the yeast transcription factor Rap1 invades and remodels chromatin. Using a reconstituted chromatin system replicating yeast promoter architecture, we demonstrate that Rap1 can bind nucleosomal DNA within a chromatin fiber but with shortened dwell times compared to naked DNA. Moreover, we show that Rap1 binding opens chromatin fiber structure by inhibiting inter-nucleosome contacts. Finally, we reveal that Rap1 collaborates with the chromatin remodeler RSC to displace promoter nucleosomes, paving the way for long-lived bound states on newly exposed DNA. Together, our results provide a mechanistic view of how Rap1 gains access and opens chromatin, thereby establishing an active promoter architecture and controlling gene expression.

## Highlights

• The yeast transcription factor Rap1 can invade compact chromatin
• Rap1 directly opens chromatin structure by preventing nucleosome stacking
• Stable Rap1 binding requires collaboration with RSC to shift promoter nucleosomes

## Graphical Abstract

**Figure:**
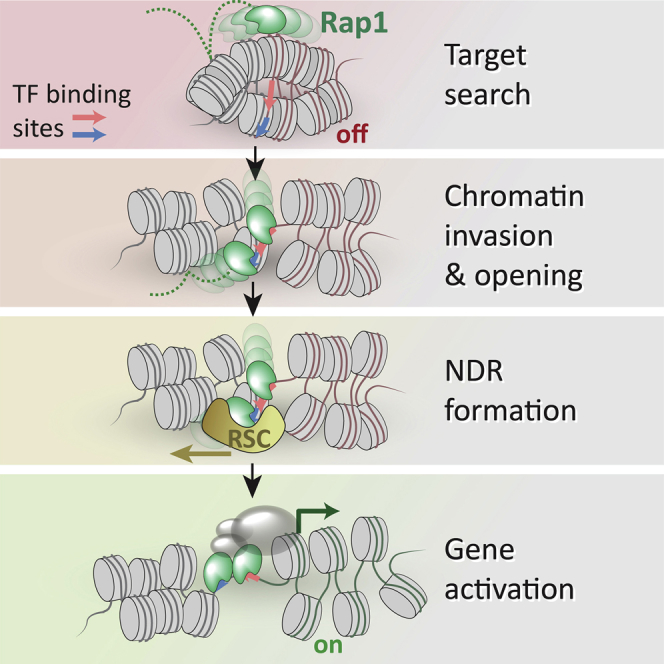

## Teaser

Mivelaz et al. use single-molecule fluorescence approaches and highly defined chromatin systems to show that the yeast transcription factor Rap1 can invade compact chromatin fibers and directly open chromatin structure. For stable binding, Rap1 then collaborates with RSC to displace nucleosomes from its binding sites, generating an active promoter state.

## Introduction

Chromatin acts as a barrier for DNA binding proteins, including transcription factors (TFs), restricting both their target search and binding-site recognition (Adams and Workman, 1995, Mirny, 2010). A subset of transcription factors named “pioneer transcription factors” (pTFs) can invade compact chromatin domains (Zaret and Mango, 2016). They then initiate chromatin structure opening (Cirillo et al., 2002, Fakhouri et al., 2010), which can coincide with linker histone loss (Iwafuchi-Doi et al., 2016) or nucleosome removal (Jin et al., 2009, Knight et al., 2014, Suto et al., 2000). Such remodeled chromatin is accessible to subsequent non-pioneer TFs (Cirillo et al., 2002), which together produce changes in transcriptional programs (Soufi et al., 2015, Zaret and Carroll, 2011).

A common feature of DNA binding domains (DBDs) of pTFs is their ability to bind partial sequence motifs displayed on nucleosomes (Soufi et al., 2015). The presence of nucleosomes may therefore have limited effects on both on-rates and residence times of pTFs. Beyond the nucleosome, higher-order chromatin structure further constrains DNA conformation and TF accessibility (Poirier et al., 2008). Indeed, high-resolution structural studies on reconstituted chromatin revealed that local structural elements, such as tetranucleosome units, form the basis of chromatin fiber organization (Schalch et al., 2005). Genomic studies have confirmed the prevalence of tetranucleosome contacts *in vivo* (Hsieh et al., 2015, Risca et al., 2017). Neighboring tetranucleosome units can interact and form fiber segments with two intertwined stacks of nucleosomes (Li et al., 2016, Schalch et al., 2005, Song et al., 2014). It is not well understood how pTFs search DNA sequences within such compact chromatin and how they invade and subsequently remodel chromatin structure.

**Figure 1:**
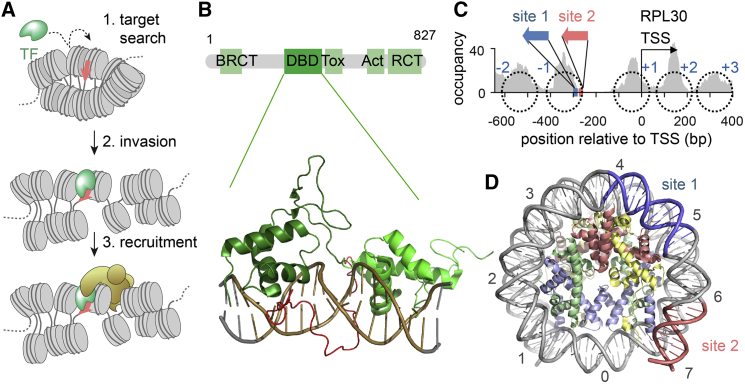
Rap1 as a Pioneer Factor in Budding Yeast (A) Scheme of pTF function: following target search (1), the pTF invades compact chromatin (2), opens chromatin, and recruits the transcription machinery (3). (B) Domain organization of budding yeast Rap1 (above) and crystal structure of a Rap1:DNA complex (PDB: 3ukg; Matot et al., 2012). Act, transcription activation domain; BRCT, BRCA 1 C terminus; DBD, DNA binding domain; RCT, Rap1 C terminus; Tox, toxicity region. (C) Organization of the *RPL30* promoter. Gray, MNase-seq profile after Rap1 depletion (Kubik et al., 2015), revealing nucleosome positions in the absence of Rap1 (black dotted circles). Plotted is nucleosome occupancy reads, normalized to 10^7^ total reads. The Rap1 binding site 1 (*S1*) (high affinity) and site 2 (*S2*) (medium affinity) fall on the −1 nucleosome. (D) Promoter −1 nucleosome, showing Rap1 binding sites *S1* and *S2* (PDB: 1AOI; Luger et al., 1997). The numbers indicate super helical locations (SHLs) of the nucleosomal DNA. See also Figure S1 and Tables S1, S2, and S3.

The intrinsic dynamics within chromatin fibers might provide a potential mechanism for pTF invasion (Cuvier and Fierz, 2017). Recent studies using force spectroscopy (Li et al., 2016) or single-molecule Förster resonance energy transfer (FRET) (Kilic et al., 2018b) revealed conformational dynamics in chromatin fibers from microseconds to seconds. It is thus conceivable that pTFs exploit fiber dynamics to invade compact chromatin, where they then recruit additional cellular machinery to enact necessary conformational reorganization to alter gene expression (Figure 1A).

Here, we test this hypothesis and reveal the mechanism of chromatin invasion, target binding, and chromatin remodeling of the pTF Rap1 (repressor activator protein 1). Rap1 is a general regulatory factor (GRF) of transcription in budding yeast (Knight et al., 2014). It has multiple roles, including the transcriptional regulation of around 5% of yeast genes (Lieb et al., 2001), repression of noncoding transcripts (Challal et al., 2018, Wu et al., 2018), and the maintenance of telomeric integrity (Wellinger and Zakian, 2012). The Rap1 DNA binding domain (DBD) consists of dual Myb-type domains connected by a short unstructured linker (König et al., 1998; Figure 1B). The DBD binds a 13-bp consensus motif with high affinity (Figure S1A), only requiring direct access to one face of the DNA (Figure 1B). Rap1 can engage a single motif in multiple binding modes, involving one or both Myb domains (Feldmann and Galletto, 2014), and previous *in vitro* studies showed that Rap1 can bind nucleosomes (Rossetti et al., 2001). In the cell, Rap1 target sites are located within nucleosome-depleted regions (NDR) upstream of the transcription start site (TSS) or within the −1 nucleosome at the two most peripheral exposed DNA major grooves (Koerber et al., 2009). A host of cell-based studies showed that Rap1 binding at these loci results in chromatin opening (Yu and Morse, 1999), nucleosome loss, and NDR formation (Badis et al., 2008, Kubik et al., 2015, van Bakel et al., 2013, Yan et al., 2018). In fact, NDRs are typical for most active eukaryotic promoters (Jiang and Pugh, 2009) and depend on the action of remodeling factors, including RSC (Badis et al., 2008, Brahma and Henikoff, 2019, Cairns et al., 1996, Hartley and Madhani, 2009, Kubik et al., 2018, Kubik et al., 2019, Ng et al., 2002, Parnell et al., 2008), SWI/SNF (Rawal et al., 2018, Yen et al., 2012), and INO80 (Krietenstein et al., 2016).

An important gene family co-regulated by Rap1 is ribosomal protein genes. Rap1 binds to the promoter/enhancer regions of >90% of these genes and initiates the recruitment of additional TFs, including Hmo1, Fhl1, and Ifh1 (Knight et al., 2014). In one of the two largest categories of ribosomal protein genes (category I), two closely spaced Rap1 binding sites are situated in the NDR upstream of the TSS (Knight et al., 2014). When Rap1 is depleted, its binding sites are covered by a stable nucleosome (Kubik et al., 2015). Digestion of yeast chromatin with limited amounts of micrococcal nuclease (MNase) followed by sequencing (MNase-seq) (Zentner and Henikoff, 2012) revealed that many NDRs contain MNase-sensitive particles (Henikoff et al., 2011, Kent et al., 2011, Weiner et al., 2010, Xi et al., 2011), which may correspond to destabilized promoter nucleosomes (Brahma and Henikoff, 2019, Chereji et al., 2017, Kubik et al., 2015, Kubik et al., 2017, Kubik et al., 2018). In category I promoters, such MNase-sensitive nucleosome-like particles appear upstream of the +1 nucleosome, co-existing with bound Rap1 (Kubik et al., 2015). Taken together, Rap1 is thus a well-characterized factor that directly impacts chromatin organization at key genes. However, the molecular mechanism by which Rap1 finds its target in compacted chromatin and how it subsequently opens chromatin and destabilizes or displaces promoter nucleosomes is not understood.

To reveal dynamic Rap1 invasion mechanisms, we reconstituted nucleosomes and chromatin fibers containing Rap1 binding sites in the configuration found in category I promoters. We find that residence times, but not binding rates, of Rap1 are strongly reduced by the presence of nucleosomes and chromatin fibers. We show that Rap1 binding alone does not disrupt or decidedly alter nucleosome conformation. In contrast, single-molecule FRET measurements reveal that Rap1 locally opens chromatin fiber structure. Finally, we demonstrate that Rap1 collaborates with RSC to displace nucleosomes from its target sites. The remodeled chromatin structure then provides an opening for stable Rap1 binding, access to further transcription factors, and finally gene regulation.

## Results

### Rap1 Binds to Nucleosomes via Nonspecific and Specific DNA Interactions

To investigate the mechanism of Rap1 nucleosome binding, we chose the ribosomal protein L30 (*RPL30*) promoter (category I) as our model system (Figure 1C). We mapped the position of the −1 nucleosome, which contains two Rap1 binding sites and is displaced *in vivo* upon Rap1 binding by MNase-seq under Rap1-depleted conditions (Figure 1C; Kubik et al., 2015). Within this nucleosome, the Rap1 binding site 1 (*S1*) is located near super helical location (SHL) 4.5, whereas site 2 (*S2*) resides near the DNA entry-exit site at SHL 6.5 (Figure 1D). Importantly, Rap1 exhibits different affinities for the two sites with a dissociation constant K_D_ of ∼10 nM for *S1* and ∼30 nM for *S2* (as determined by electromobility shift assays [EMSAs]; Figures S1C–S1E). *In vivo*, both sites contribute to the expression of the *RPL30* gene product (Knight et al., 2014).

**Figure 2:**
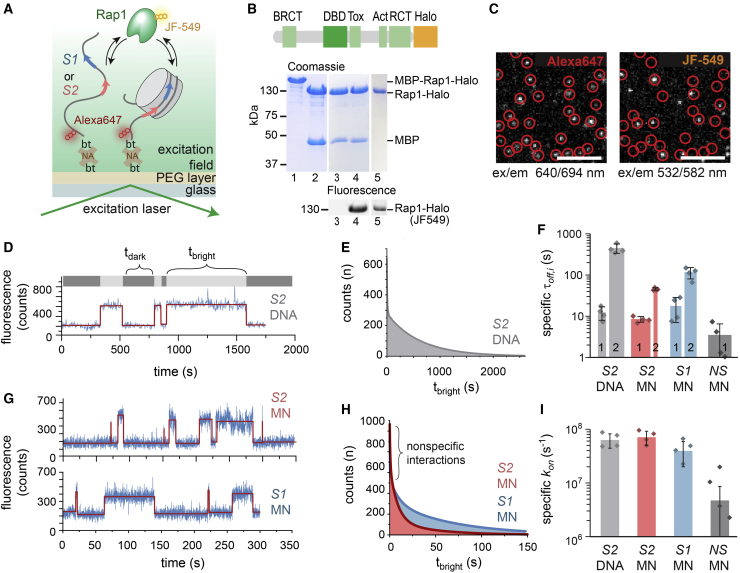
Rap1 Recognizes Target Sites within Nucleosomal DNA (A) Scheme of the smTIRFM experiment to detect Rap1 binding to *S1*- or *S2*-containing, Alexa-Fluor-647-labeled, and immobilized DNA or nucleosomes. bt-NA, biotin-neutravidin. (B) Expression and labeling of Rap1. Lanes: (1) purified MBP-Rap1-Halo; (2) MBP cleavage; (3 and 4) before and after JF-549 labeling; and (5) purified Rap1. (C) Representative smTIRF images showing nucleosome positions in the far-red channel (left, red circles) and Rap1 binding events in the green-orange channel (right). Scale bars: 5 μm; ex, excitation wavelength; em, emission wavelength. (D) Representative fluorescence time trace of Rap1 binding events to *S2* containing naked DNA, detected by JF-549 emission. The trace was fitted (red), and *t*_*dark*_ and *t*_*bright*_ were determined by a thresholding algorithm. (E) Cumulative histogram of Rap1 binding intervals (*t*_*bright*_) on *S2* DNA fitted by a 2-exponential function $y=\sum_{i=1}^{2}A_{i}\;\exp\left(-t/\tau_{off,\;i}\right)$ (solid line). For all fit results, see Table S4. (F) Specific dissociation time constants (τ_off,i_ > 1 s) of Rap1 for *S2* DNA, *S1* and *S2* containing mononucleosomes (MN), or nucleosomes lacking a binding site (NS), uncorrected for dye photobleaching. The width of the bars indicates the percentage of events associated with the indicated time constants (i.e., amplitudes *A*_*i*_ of the multi-exponential fits shown in E and H). n = 4 to 5; error bars: SD. (G) Representative fluorescence time trace of Rap1 binding events to *S1* (bottom) and *S2* (top) containing MNs. The data were analyzed as in (D). (H) Cumulative histogram of Rap1 binding intervals (*t*_*bright*_) on *S1*- and *S2*-containing MNs fitted by a 3-exponential function $y=\sum_{i=0}^{2}A_{i}\;\exp\left(-t/\tau_{off,\;i}\right)$ (solid line). (I) Specific on-rate constants (*k*_*on*_*= 1/*τ_*on*_) for all species obtained from a single-exponential fit to cumulative histograms of *t*_*dark*_ values and corrected for the contribution from nonspecific interactions (STAR Methods). See also Figures S2 and S3 and Tables S1, S2, S3, and S4.

We then implemented a single-molecule total internal reflection fluorescence microscopy approach (smTIRFM) to directly observe dynamic Rap1 binding to promoter nucleosomes via fluorescence colocalization (Figure 2A; Kilic et al., 2015). We first generated a 235-bp DNA template based on the 601 nucleosome positioning sequence (Lowary and Widom, 1998), which contained one or both Rap1 binding sites, *S1* or *S2*, at the same position as in the native −1 promoter nucleosome (Figures 1D and S1B; Tables S1, S2, and S3). Moreover, the DNA constructs contained a far-red fluorescent dye (Alexa Fluor 647) and a biotin moiety for immobilization. We then used this DNA directly for measurements or reconstituted nucleosomes using recombinantly expressed histones (Figures 2A and S2A–S2E). Second, we purified full-length Rap1 as a Halo-tag fusion from insect cells and fluorescently labeled the protein with the highly photostable green-orange dye JF-549 (Grimm et al., 2015; Figures 2B and S2F–S2K). Labeled Rap1 exhibited similar DNA binding compared to published values (Knight et al., 2014; Figures S1C–S1E).

Having all components in hand, in a first set of experiments, we immobilized *S1*- or *S2*-containing naked DNA strands in a microfluidic channel and determined their position by smTIRFM imaging in the far-red channel (Figure 2C). We then injected Rap1 at a concentration chosen such that individual, non-overlapping binding events could be detected as fluorescent spots in the green-orange channel (usually 50–100 pM). Colocalization of Rap1 with DNA positions indicated binding (Figure 2C). We then recorded movies that revealed the binding kinetics of Rap1 to *S1*- or *S2*-containing naked DNA. For each DNA molecule, extracted kinetic traces allowed us to determine the length of individual binding events (*t*_*bright*_) and intermittent search times (*t*_*dark*_). The effect of dye photobleaching on residence time measurements was reduced by stroboscopic imaging (Figure S3A).

Although dynamic Rap1 binding was observed for the medium affinity site *S2* (Figure 2D), individual binding events to the high-affinity site *S1* were so long (>40 min) that we were not able to obtain suitable statistics (Figure S3B). For *S2*-containing DNA, we constructed cumulative lifetime histograms of bright times (*t*_*bright*_) (Figure 2E), which were fitted using a bi-exponential function, yielding two residence times τ_off,1_ and τ_off,2_ (Figure 2F; see Table S4 for all rate constants). Of all binding events, 35% exhibited a short residence time (τ_off,1_ = 12.4 ± 4.5 s), whereas the remaining 65% showed slow Rap1 dissociation kinetics (τ_off,2_ = 452 ± 115 s). Due to the dual Myb-type DBD, these different residence times may indicate different binding modes where either the whole or only a partial DNA binding motif is engaged. Under equilibrium binding conditions, Rap1 thus forms long-lived complexes with free DNA, resulting in residence times in the minutes to hours range for *S1* and *S2*.

In contrast, the presence of mononucleosomes (MNs) shortened the residence time of Rap1, as observed in kinetic traces for MNs containing either *S1* or *S2* (Figure 2G) and in the corresponding lifetime histograms (Figure 2H). Here, a tri-exponential function was required to describe the data (Figures S3C–S3E). Around 50% of all detected events were short lived, with a time constant of 0.2 < τ_off,0_ < 0.7 s. We attribute these fast events to nonspecific interactions of Rap1 with the nucleosomal DNA. Specific Rap1 binding to *S1* or *S2* further resulted in two longer time constants τ_off,1_ and τ_off,2_. Rap1 binding to the high-affinity site *S1* was associated with longer residence times (τ_off,1_ = 18 ± 11 s and τ_off,2_ > 100 s) compared to *S2* (τ_off,1_ = 8.4 ± 1.4 s; τ_off,2_ = 46 ± 3 s; Figure 2F). This was not necessarily expected, as *S1* is located further within the nucleosome and thus potentially less accessible than *S2*, which resides at the DNA entry-exit site. To test the effect of site positioning, we moved *S2* from SHL 6.5 to SHL 4.5 and observed an additional reduction in Rap1 residence time to τ_off,1_ = 2.4 ± 0.4 s and τ_off,2_ = 7.7 ± 1.9 s (Figures S3F–S3H). This observation suggests that a lower number of histone contacts and increased conformational fluctuations of the DNA at SHL 6.5 render this Rap1 site locally more accessible and thus allow higher-affinity binding. Of note, having both sites *S1* and *S2* in the same nucleosome resulted in a superposition of the individual binding kinetics under our measurement conditions (Figures S3I and S3J).

Specific binding rates (*k*_*on*_) obtained from analyzing lifetime histograms of dark times (*t*_*dark*_) (Figures S3K–S3M) were comparable for all analyzed DNA and nucleosome constructs (Figure 2I). This demonstrates that the Rap1 target search kinetics are not strongly influenced by the presence of nucleosomes.

Finally, we also probed Rap1 binding to nucleosomes without binding sites (Figures S3N–S3P). A majority (83%) of all detected binding events were shorter than 1 s although the remaining 17% persisted for only 3.5 ± 3 s, consistent with nonspecific nucleosome interactions (Figures 2F and 2I). Together, these results indicate that Rap1 can bind to nucleosomal DNA, with overall similar on-rates and with reduced residence times (>10-fold) compared to free DNA, which depend on the site’s position on the nucleosome.

### Chromatin Structure Shortens Rap1 Dwell Times

**Figure 3:**
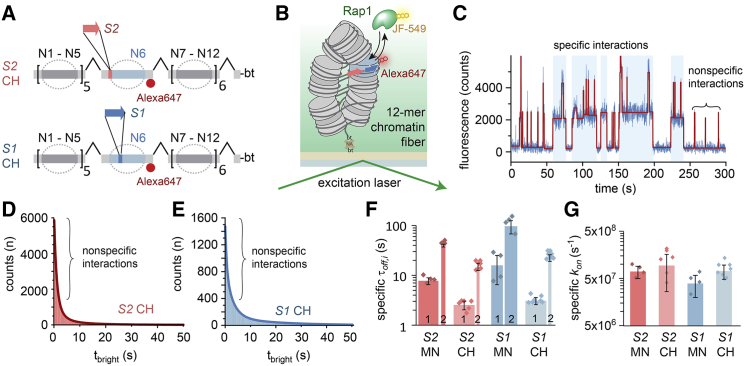
Chromatin Higher-Order Structure Reduces Rap1 Dwell Time (A) Scheme of DNA preparation used to introduce Rap1 target sites *S1* and *S2* into the central nucleosome (N6) of a chromatin fiber (CH). (B) Scheme of the smTIRFM experiment to measure Rap1 binding kinetics in a chromatin fiber context. (C) Representative fluorescence time trace of Rap1 binding events to *S1*-containing chromatin arrays. The trace is fitted (red); t_dark_ and t_bright_ were determined by a thresholding algorithm. (D) Cumulative histogram of Rap1 binding intervals (t_bright_) to chromatin fibers, containing *S1* fitted by a 3-exponential function (solid line). For all fit results, see Table S4. (E) Cumulative histogram of Rap1 binding to chromatin arrays, containing *S2* fitted by a 3-exponential function (solid line). (F) Specific binding time constants (τ_off,i_ > 1 s) of Rap1 for S1 in a nucleosome (MN) versus chromatin fiber (CH) and S2 MN versus CH. The widths of the bars indicate the percentage of events associated with the indicated time constants (i.e., amplitudes *A*_*i*_ of the multi-exponential fits shown in D and E). n = 4 to 5; error bars: SD. (G) Specific on-rate constants (*k*_*on*_*= 1/*τ_*on*_) for MNs and CHs containing *S1* and *S2*, obtained from a single-exponential fit to cumulative histograms of *t*_*dark*_ values and corrected for the contribution from nonspecific interactions (STAR Methods). See also Figure S4 and Tables S1, S2, S3, and S4.

In cells, pTFs invade compact chromatin structure, which has been shown to reduce overall TF accessibility (Soufi et al., 2012). We therefore proceeded to investigate the mechanism of chromatin invasion by Rap1. We employed a modular system to construct chromatin fibers (Kilic et al., 2018b), based on a 12-mer repeat of 601 nucleosome positioning sequences, each separated by 30 bp of linker DNA. We assembled two chromatin fiber types, containing Rap1 target sites *S1* or *S2* in their central nucleosome (N6) in the same orientation as in the *RPL30* promoter (Figures 3A and S4A–S4H). The chromatin fibers were then immobilized in a flow cell, and Rap1 binding dynamics were determined using smTIRFM (Figure 3B).

Under our measurement conditions, chromatin fibers exist in a compact state (Allahverdi et al., 2015). Compared to MNs, we observed an increase in short (0.6-s) Rap1 binding events on chromatin fibers (∼70% of all detections; Figure 3C), which can be attributed to nonspecific probing interactions. Rap1 thus rapidly samples the chromatin fiber in its search for target sites. For *S1*- or *S2*-containing fibers (but not for chromatin devoid of such; Figures S4I–S4K), we detected additional longer-lived binding events (Figure 3C). Rap1 can thus invade compact chromatin fibers. Analyzing the lifetime histograms (Figures 3D and 3E) revealed two longer time constants, corresponding to specific interactions (Figures S4L–S4N). This is similar to the situation in MNs, reflecting multiple Rap1 binding modes. The Rap1 residence times in chromatin fibers were, however, further reduced (Figure 3F) by about 3-fold for *S2* (τ_off,1_ = 2.6 ± 0.6 s; τ_off,2_ = 16.8 ± 3 s) and 5-fold for *S1* (τ_off,1_ = 3.2 ± 0.6 s; τ_off,2_ = 25.6 ± 4.0 s) compared to MNs. This shortening of Rap1 dwell times demonstrates that chromatin fiber structure acts as an additional hindrance to Rap1 binding.

To determine whether chromatin inhibits the target search process of Rap1, we compared *k*_*on*_ values between DNA, MNs, and chromatin fibers. We could not detect any significant differences between the systems (Figure 3G). It is thus conceivable that reduced access in fibers is balanced by a more efficient search process, as Rap1 can hop or slide along chromatin in search of its binding site, using nonspecific DNA interactions as a means of chromatin anchoring. Chromatin dynamics on the millisecond timescale (Kilic et al., 2018b) will eventually expose internal DNA sites, allowing the factor to bind to its target sequence with similar kinetics compared to naked DNA.

### Rap1 Binding Does Not Evict or Distort Bound Nucleosomes

**Figure 4:**
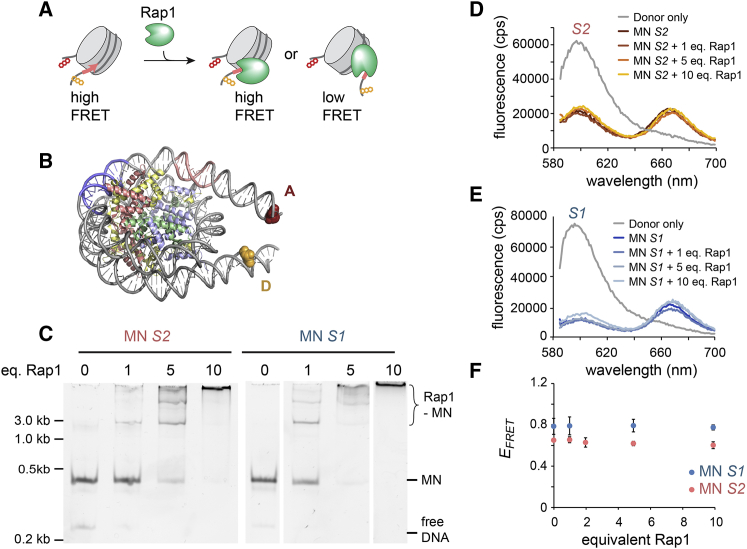
Rap1 Does Not Open Nucleosome Structure (A) Scheme of experiment to detect nucleosome structure change due to Rap1 binding by FRET. (B) Nucleosome structure (PDB: 1AOI) showing attachment points of FRET probes. (C) EMSA showing Rap1 binding to *S1* and *S2* nucleosomes at indicated concentration equivalents (eq.). Lanes were re-arranged for clarity. (D) Fluorescence spectra for *S2* nucleosome in complex with indicated equivalents of Rap1. (E) Fluorescence spectra for *S1* nucleosome in complex with indicated equivalents of Rap1. (F) FRET efficiency calculated for *S2* and *S1* nucleosomes as a function of equivalents added Rap1. Error bars: SD; n = 2. See also Figure S5.

Having established that Rap1 indeed binds to nucleosomes and can invade chromatin structure, we wondered whether Rap1 can remodel chromatin, i.e., by directly opening chromatin structure (Zaret and Carroll, 2011). In cells, Rap1 binding results in the destabilization and disruption of promoter nucleosomes (Knight et al., 2014, Kubik et al., 2015, van Bakel et al., 2013, Yan et al., 2018), paving the way for binding of subsequent TFs and establishing a chromatin state permissive to transcription. First, we wondered whether Rap1 can directly destabilize nucleosomes, leading to DNA unwrapping as observed for other TFs (Donovan et al., 2019, Li et al., 2005, Li and Widom, 2004, Luo et al., 2014). We therefore established a FRET-based assay to monitor nucleosomal DNA unwrapping (Figures 4A and S5A–S5D). We positioned FRET donor (Alexa Fluor 568) and acceptor (Alexa Fluor 647) dyes within the linker DNA of *S1*- and *S2*-containing nucleosomes, such that partial DNA unwrapping (or nucleosome disassembly) will lead to FRET loss (Figures 4A, 4B, and S5D). Rap1 (1–10 equivalents) bound to nucleosomes as judged by EMSA (Figure 4C). However, no change in FRET efficiency (*E*_*FRET*_) was observed for both *S1* and *S2* nucleosomes (Figures 4D and 4E), even at the highest Rap1 concentrations (Figure 4F). These experiments demonstrate that Rap1 binding to *S1* or *S2* does not dramatically affect nucleosome structure and does not result in either DNA unwrapping or histone loss. We further measured Rap1 binding affinities when its target motif is moved in 3-bp steps around the nucleosomal DNA helix. Indeed, Rap1 bound to *S1* with a K_D_ of ∼80 nM, with ∼50 nM for *S1* shifted by 3 bp and ∼60 nM for *S1* shifted by 6 bp (Figures S5E–S5G). These rotational affinity changes are consistent with Rap1 binding to DNA displayed on the nucleosome surface. The differences are, however, small, pointing toward a role of local DNA flexibility in leveling the binding energy landscape.

Importantly, nucleosomes formed using the native *RPL30* DNA sequence also remained stable upon Rap1 binding (Figures S5H–S5J). Although *RPL30* nucleosomes yielded overall lower FRET values compared to 601 derived sequences (as the nucleosomes were less well positioned), Rap1 binding did not result in FRET loss (Figures S5K–S5M). Finally, single-molecule Rap1 binding experiments using *RPL30* nucleosomes containing site *S1* revealed comparable residence times to 601 nucleosomes (Figures S5N–S5Q), and no progressive loss of nucleosomes was observed (Figure S5P). Together, these experiments demonstrate that Rap1 binding itself does not greatly distort or disrupt nucleosome structure.

### Rap1 Locally Opens Chromatin Structure

**Figure 5:**
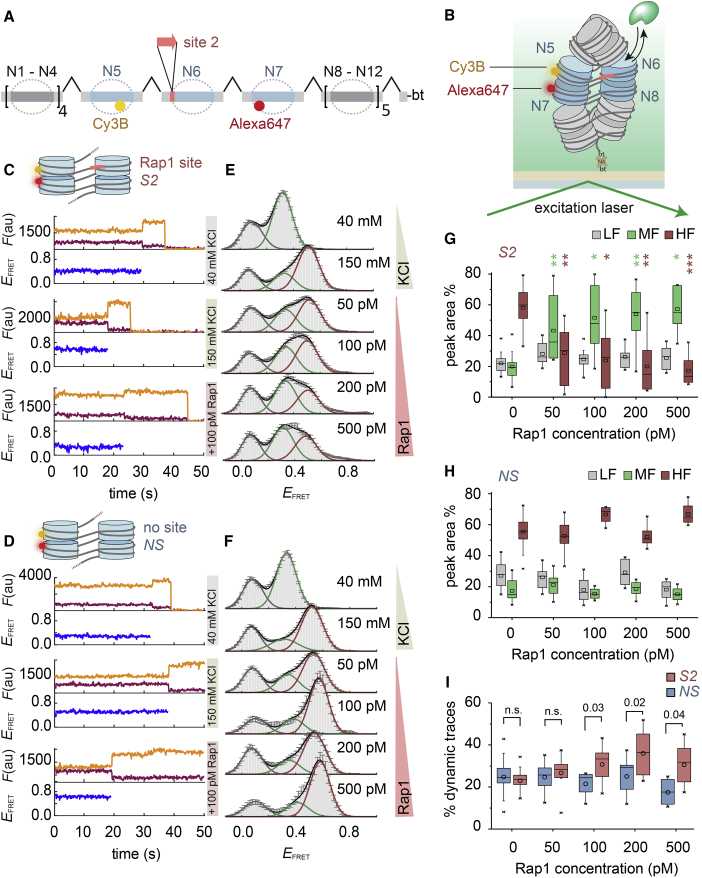
Chromatin Remodeling Induced by Rap1 Invasion as Observed by smFRET (A) Scheme of chromatin DNA assembly to introduce a Rap1 site at nucleosome N6, as well as a FRET donor (Cy3B, yellow) and acceptor (Alexa Fluor 647, red) at nucleosomes N5 and N7. (B) Scheme of a smFRET-TIRF experiment. (C) Individual kinetic traces of donor (orange) and acceptor (red) fluorescence emission and FRET efficiency (*E*_*FRET*_, blue) for chromatin fibers containing *S2* at the indicated KCl and Rap1 concentrations. All Rap1 experiments were performed at 150 mM KCl. (D) Similar to (C) but for chromatin lacking Rap1 binding sites (*NS*). (E) Histograms of *E*_*FRET*_ of *S2*-containing chromatin fibers at the indicated KCl and Rap1 concentrations. All Rap1 experiments were performed at 150 mM KCl. Histograms were fitted by Gaussian functions, revealing a low-FRET (LF) (gray), medium-FRET (MF) (green), and high-FRET (HF) (red) population. Error bars are SEM; for the number of traces and parameters of Gaussian fits, see Tables S5 and S6. (F) Similar to (E) but for chromatin lacking Rap1 binding sites (*NS*). (G) Percentage of each FRET sub-population, LF, MF, and HF for chromatin containing *S2*. Box: 25–75 percentiles; whiskers: outliers (factor 1.5); line: median; open symbol: mean. For number of experiments, see Table S6. ^∗^10^−3^ > p < 10^−4^; ^∗∗^10^−4^ > p < 10^−5^; ^∗∗∗^p < 10^−5^, two-tailed Student’s t test between peak area % of LF, MF, and HF populations for *S2* or *NS* nucleosomes (see H). (H) Similar to (G) but for chromatin lacking Rap1 binding sites (*NS*). (I) Percentage of dynamic traces for *S2* and *NS* chromatin. Box: similar to (H). For the identification of dynamic traces, see STAR Methods. p: two-tailed Student’s t test; n.s.: p > 0.05. See also Figure S6 and Tables S5 and S6.

Although the structure of individual nucleosomes is not disrupted by Rap1 binding, higher-order chromatin structure might be altered. We thus performed single-molecule FRET (smFRET) experiments that directly report on nucleosome stacking interactions (Kilic et al., 2018a, Kilic et al., 2018b). We flanked the *S2*-containing central nucleosome (N6) of a 12-mer chromatin fiber by nucleosomes carrying a FRET donor (Cy3B in N5) and acceptor dye (Alexa Fluor 647 in N7; Figures 5A and S6A–S6G). As a control, we also produced fibers without a binding site (no site [*NS*]).

First, we characterized the conformations exhibited by these chromatin fibers by measuring FRET efficiency (*E*_*FRET*_*)*, which reports on the inter-nucleosome distance. High *E*_*FRET*_ values indicate compact chromatin, whereas a reduction in *E*_*FRET*_ reveals a loss in higher-order structure (e.g., due to unstacking of tetranucleosome units). We immobilized fibers in a flow channel and recorded movies under smTIRF conditions (Figure 5B). From the resulting time traces (Figures 5C and 5D), we constructed FRET histograms, which were approximated by a sum of 3 Gaussian functions (Figures 5E and 5F). At native ionic strength (150 mM KCl), we observed a major population at high FRET (HF) (E_FRET_ ∼0.5) and minor populations at medium (MF) (E_FRET_ ∼0.3) and low FRET (LF) (E_FRET_ < 0.1; Figures 5C–5F). Similar results were obtained in the presence of divalent cations (4 mM Mg^2+^; Figures S6H–S6O; Dorigo et al., 2003). In contrast, at low ionic strength (40 mM KCl), where chromatin is open, the HF population was absent and only the MF state was observed. Together, these measurements enabled us to assign the HF state to compact chromatin and nucleosome stacking, whereas the MF state reflects open chromatin. The LF state is observed for all fibers and most probably indicates chromatin assembly defects (e.g., shifted or lacking nucleosomes at dye positions; Kilic et al., 2018b).

We then titrated Rap1 to chromatin fibers with (*S2*) or without (*NS*) a Rap1 binding site, using concentrations from 50 to 500 pM. For *S2*-containing fibers, the fraction of tightly compacted chromatin (the HF population) was reduced and locally opened chromatin (MF) was populated with increasing Rap1 concentration (Figures 5E and 5G). In contrast, chromatin lacking Rap1 binding sites was not sensitive to Rap1 addition (Figures 5F and 5H). Moreover, a subset (∼18%–25%) of FRET traces exhibited anti-correlated fluctuations in the donor and acceptor fluorescence channels, indicative of conformational dynamics on the second timescale (Figures S6P and S6Q). Rap1-dependent chromatin opening for *S2*, but not for *NS*, was associated by an increase in the subset of traces exhibiting such conformational fluctuations (31%–37%; Figure 5I). This directly indicates that Rap1 samples compact chromatin and invades chromatin structure, most probably by exploiting intrinsic chromatin fiber dynamics. Once bound, local higher-order structure is disrupted by the pTF, thereby enabling chromatin access for subsequent factors.

### Rap1 Collaborates with RSC to Displace Promoter Nucleosomes

**Figure 6:**
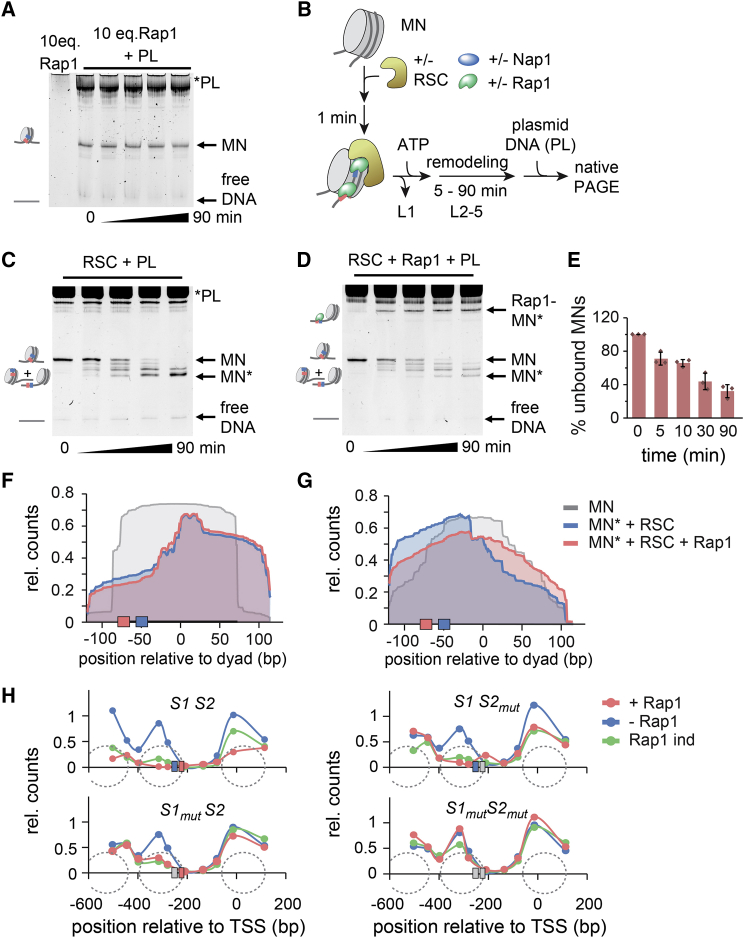
RSC Enables Stable Rap1 Binding by Exposing Binding Sites (A) Native PAGE analysis of Rap1 binding for indicated times followed by incubation with competitor plasmid DNA (PL). L1 and L2–L5: lanes in (A) and (C) and (D). (B) Scheme of RSC remodeling assay. Note that Nap1 is not strictly required in these experiments (Figure S7C). (C) Native PAGE analysis of remodeling assays; MN^∗^, remodeled mononucleosome. (D) Native PAGE analysis of remodeling assays in the presence of 10 eq. of Rap1. (E) Integrated unbound nucleosome bands from (D) (n = 3; error bars SD). (F) MNase-seq results from RSC remodeling assays for 601 nucleosomes (P3_S1S2). Gray, nucleosome start position; blue, RSC remodeling for 90 min in absence of Rap1; red, RSC remodeling for 90 min in presence of 10 eq. Rap1. Shown are reads normalized to number of total reads. (G) Same as in (F) but for *RPL30* nucleosomes (P3_RPL30). (H) Effect of Rap1 binding on nucleosome stability at the *RPL30* promoter in yeast. Nucleosome positions were determined using qPCR after MNase digestion of chromatin. Promoters analyzed contained both Rap1 binding sites (*S1S2*), *S1* mutated (S1_mut_S2), *S2* mutated (S1S2_mut_), or both binding sites mutated (S1_mut_S2_mut_). Data shown are for cells where Rap1 is present (Rap1+, red), Rap1 has been depleted from the nucleus for 1 h by anchor-away (Rap1−, blue), and where Rap1 has been re-introduced for 2 h following depletion by expressing a *RAP1* construct from an inducible promoter (Rap1 ind, green). See also Figure S7 and Table S7.

Taken together, our biophysical analyses show that Rap1 increases accessibility within compact chromatin fibers but does not unwrap or evict bound nucleosomes. Moreover, Rap1 exhibits short residence times on nucleosomal DNA but is more stably bound within naked DNA, e.g., as a result of nucleosome shifting. The amount of such stably bound Rap1 can be assessed by incubating *S1S2*-containing nucleosomes with Rap1 over time (0–90 min) at 30°C. In the presence of excess Rap1, nucleosomes can no longer be analyzed on native gels, due to short-lived and nonspecific Rap1 binding (Figure 6A, lane 1). We thus added an excess of competitor plasmid (PL) that acts as a sink for all nonspecifically or dynamically bound proteins. Following this protocol, all Rap1 is quickly competed off, and no Rap1-bound nucleosomes were detected by native PAGE irrespective of incubation time (Figure 6A, lanes 2–6). Rap1 by itself is thus not able to shift nucleosomes or liberate its target sites, which would allow stable long-lived binding.

In yeast, the RSC complex is involved in the formation and maintenance of nucleosome-free regions within promoters (Badis et al., 2008, Brahma and Henikoff, 2019, Cairns et al., 1996, Hartley and Madhani, 2009, Kubik et al., 2015, Kubik et al., 2018, Ng et al., 2002, Parnell et al., 2008) and plays an important role in the organization of ribosomal protein gene promoters (Kubik et al., 2015, Kubik et al., 2018). We therefore hypothesized that a remodeler, such as RSC, could collaborate with Rap1 to clear promoters as observed *in vivo*.

We used purified RSC complex (Kurat et al., 2017) to perform remodeling assays (Clapier et al., 2016, Lorch et al., 2006; Figure 6B). In the absence of Rap1, RSC slid nucleosomes to a peripheral DNA position (Figures 6C and S7A–S7C). When performing these experiments in the presence of Rap1, the repositioned nucleosomes were stably bound by Rap1, as judged by the disappearance of the nucleosomal band (Figure 6D; quantified in Figure 6E) and the appearance of a new species. Using nucleosomes containing fluorescently labeled H2A, either in concert with RSC or by directly reconstituting end-shifted nucleosomes in the absence of RSC, we could clearly identify these new species as Rap1-nucleosome complexes (Figures S7D and S7E). These results thus show that nucleosomes remodeled by RSC provide a stable binding environment for Rap1.

Intriguingly, when RSC remodeling and Rap1 binding were performed sequentially, stable Rap1 nucleosome binding was reduced (Figure S7F). This indicated that Rap1 might collaborate with RSC by biasing the directionality of the remodeling reaction. We thus performed RSC remodeling experiments (with or without Rap1) on both 601-based (*S1S2*) or *RPL30* nucleosomes (Figure S7G) and mapped nucleosome positioning using MNase-seq.

In the absence of Rap1, RSC shifted nucleosomes based on 601 DNA (initially positioned in the DNA center; gray profile in Figure 6F) primarily to the DNA end distal to the Rap1 binding sites (blue profile in Figure 6F). In contrast, *RPL30* nucleosomes (Figure 6G, gray profile) were preferentially shifted toward the Rap1 sites (blue profile in Figure 6G). Such sequence-dependent remodeling by RSC has been described before and is imparted by Rsc3 binding motifs (i.e., variants of CGCG), of which several exist within the 601 sequence, and poly-A tracts, which are present within *RPL30* (Badis et al., 2008, Krietenstein et al., 2016, Kubik et al., 2015, Kubik et al., 2018).

Remodeling reactions in the presence of Rap1 resulted in an altered positional distribution of the nucleosomes on the DNA. In the 601 context, Rap1 could further reduce the nucleosome footprint overlapping with *S1* and *S2* and stably bind DNA, which was liberated by RSC (red profile in Figure 6F). In *RPL30* nucleosomes, Rap1 showed a more pronounced effect, reducing RSC-catalyzed nucleosome encroachment over its binding motifs (red profile in Figure 6G). Together, these experiments show that Rap1 can bias RSC remodeling, resulting in the clearance of nucleosomes from promoter sequences.

Finally, we analyzed whether such Rap1-coupled dynamic nucleosome repositioning can be observed in living yeast. We generated a yeast strain carrying a reporter plasmid bearing the *RPL30* promoter. Nucleosome positioning on this test promoter was probed by MNase treatment followed by fragment mapping using qPCR (Knight et al., 2014). If at least one functional Rap1 binding motif was present, Rap1 was stably bound, the −1 promoter nucleosome was displaced (Figure 6H), and the reporter gene was expressed (Figures S7H–S7L). In contrast, if both Rap1 binding sites were mutated, a nucleosome residing in the NDR was detected and reporter gene expression was abolished (Figures 6H and S7L). Interestingly, when Rap1 was depleted by an “anchor-away” approach (Haruki et al., 2008, Kubik et al., 2015), the −1 nucleosome was restored for all promoters within 1 h (Figure 6D). Subsequent re-induction of Rap1 finally led to rapid nucleosome displacement (<2 h; Figure 6D). Together, this demonstrates that Rap1 plays a central role in dynamically altering the local chromatin environment and determining the fate of bound nucleosomes.

## Discussion

Using a defined reconstituted chromatin system, we directly observed the chromatin invasion process on an essential yeast pTF, Rap1. This allowed us to draw the following main conclusions: first, Rap1 can bind to both nucleosomes and compact chromatin fibers, but its dwell times are reduced by higher-level chromatin organization. Conversely, target search kinetics—driven through nonspecific DNA interactions—were not affected by chromatin structure for the binding sites that we probed. Second, we found that Rap1 can access its binding sites without drastically altering nucleosome conformation. In contrast, Rap1 disrupts stacking interactions between neighboring nucleosomes in chromatin fibers, leading to an open chromatin conformation and increased local access. Third, we showed that stable Rap1 binding within chromatin requires collaboration with the RSC remodeler. This conclusion is supported by observations in live yeast cells, where nucleosomes directly targeted by Rap1 are dynamically removed. Together, these data provide a comprehensive view into how the yeast pTF Rap1 locally remodels the chromatin landscape to form NDRs at target promoters.

### Multimodal DNA Interactions Guide Rap1 Chromatin Invasion

Several features enable Rap1 to search the chromatin landscape and bind to nucleosomal DNA. First, the Rap1 DBD is embedded in flanking basic regions, which have been shown to enable nonspecific DNA binding for other TFs (Raccaud et al., 2019, Raccaud and Suter, 2018). In our single-molecule studies, we observed frequent short-lived interactions compatible with a search process driven by nonspecific DNA interactions. Second, Rap1 binds to consensus sequences with very high affinity, ranging in K_D_ from low pM (Vignais et al., 1990) to low nM (Williams et al., 2010). This is consistent with our observation of residence times on the min to h timescale on naked DNA. Third, the dual Myb domains do not completely envelop the target DNA when bound (König et al., 1998). They thus do not require unwrapping of nucleosomal DNA and allow Rap1 to bind chromatin with similar on-rates compared to naked DNA. Nevertheless, we found that Rap1 residence times on nucleosomes were reduced (albeit not nearly as much as for other TFs; Luo et al., 2014) and dependent on both the nature of the target site and the rotational positioning of the sites on the nucleosome. The reduction in dwell times most probably arises from a combination of partial binding site occlusion and from the highly bent DNA structure on the nucleosome, both known mechanisms that affect TF affinity and sequence specificity (Zhu et al., 2018). Still, due to the flexibility of its DBD, Rap1 shows significant chromatin binding, consistent with its role as a pioneer factor. This binding mechanism has been observed for mammalian pluripotency factors, such as Sox2 (Soufi et al., 2015), which can also bind partial DNA motifs in a nucleosomal context (albeit with comparable affinity compared to DNA, whereas binding of Rap1 is weakened by the presence of nucleosomes). In contrast, Reb1 and Cbf1 (budding yeast pTFs) have been shown to require partial nucleosome unwrapping for binding (Donovan et al., 2019). Interestingly, these factors compensate a reduction in on-rate by increased residence times on nucleosomal substrates. In yet another interaction mode, the mammalian pTF FoxA contains a core-histone binding motif (Cirillo et al., 2002) and a DNA-binding domain with similarities to the linker histone H1 (Clark et al., 1993, Iwafuchi-Doi et al., 2016). These motifs thus provide additional stability on chromatin substrates (Cirillo and Zaret, 1999) and open chromatin by linker-histone displacement. Similarly, the related factor FoxO1 can bind to nucleosomes and open linker-histone compacted chromatin (Hatta and Cirillo, 2007). In summary, chromatin binding and invasion is a defining feature of pTFs, but multiple mechanisms have evolved that allow different pTFs to engage chromatin.

### Rap1 Passively Alters Local Higher-Order Chromatin Structure

Chromatin fibers are conformationally heterogeneous, as exemplified by structural studies (Ekundayo et al., 2017, Garcia-Saez et al., 2018, Routh et al., 2008) or crosslinking experiments (Grigoryev et al., 2009). We and others have previously shown that chromatin fiber contacts are highly dynamic (Kilic et al., 2018b, Li et al., 2016, Poirier et al., 2009). Importantly, the basic units of chromatin organization, tetranucleosome units, exhibit dynamics on a millisecond timescale (Kilic et al., 2018b). This exposes all internal DNA sites over time, yielding opportunities for protein factors, including Rap1, to gain access. Experiments based on endonuclease digestion of chromatin fibers indicated that pTFs increase local chromatin access (Cirillo et al., 2002). Here, we directly observe chromatin fiber structure as a function of pTF invasion using smFRET between neighboring nucleosomes. Mechanistically, our results suggest that Rap1 can capture transiently exposed binding sites and then reduce or block the reformation of a closed tetranucleosome unit. This may not only increase the accessibility for other TFs but also enables binding of remodeling factors.

### RSC Is Required for NDR Generation and Stable Rap1 Binding

Extended NDRs are a prominent feature of active yeast promoters, and Rap1 is a key driver of nucleosome displacement (Ganapathi et al., 2011, Knight et al., 2014, Yu et al., 2001). Chromatin opening at Rap1-regulated promoters *in vivo* has been shown to require the Rap1 DNA binding domain (Yu et al., 2001) but is not reliant on other TFs (Ganapathi et al., 2011). However, in our studies, we found that Rap1, by itself, is not sufficient to clear a promoter region of nucleosomes.

Remodeling factors play a key role in promoter organization (Yen et al., 2012) and have been shown to be important for the activity of multiple TFs and pTFs, for example, Oct4 or GATA3, which both rely on BRG1 (King and Klose, 2017, Takaku et al., 2016) or INO80 (Wang et al., 2014), or CTCF and REST that require SNF2H and BRG1, respectively (Barisic et al., 2019). Moreover, the synthetic TF Gal4-VP16 was shown to recruit SWI/SNF to open chromatin (Gutiérrez et al., 2007). Here, we show that nucleosome displacement by RSC enables stable Rap1 binding and promotes NDR formation. For the *RPL30* promoter, mapping experiments in yeast showed that NDR formation is dependent on both RSC and Rap1, with the latter factor dominating (Kubik et al., 2018). Moreover, the presence of RSC at the *RPL30* promoter is influenced by Rap1, further indicating a collaborative function (Kubik et al., 2018). In contrast to mammalian examples that indicate direct remodeler recruitment (King and Klose, 2017), no direct interaction between RSC and Rap1 is described to date. However, RSC may be recruited indirectly or as a result of increased chromatin accessibility upon Rap1 binding.

**Figure 7:**
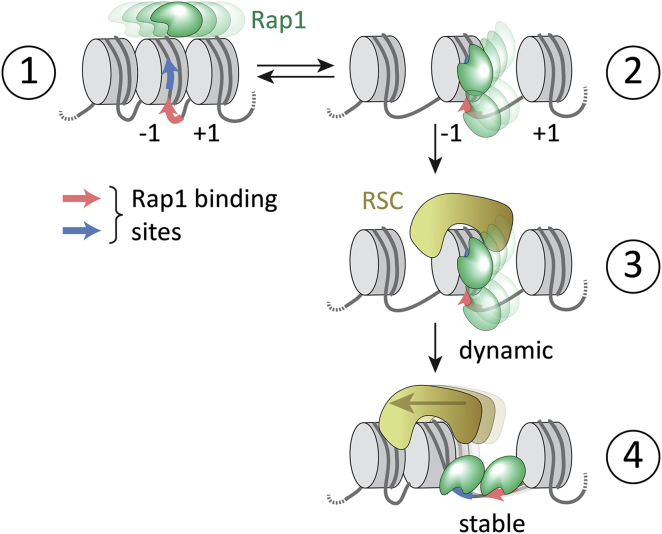
A Dynamic Model for Rap1-Mediated Promoter Chromatin Remodeling Rap1 searches chromatin (step 1) and its dynamic binding (2–25 s) to a promoter site results in local chromatin opening (step 2), where Rap1 remains dynamically bound. RSC-mediated nucleosome sliding opens the NDR and exposes the DNA containing Rap1 binding sites (step 3). The fully exposed binding sites allow stable Rap1 binding (step 4) with long residence times (free DNA τ_res_ > 450 s) and prevent further nucleosome encroachment.

The directionality of RSC remodeling is controlled by DNA sequence, in particular by poly-dA tracts and GC-rich motifs (Badis et al., 2008, Krietenstein et al., 2016, Kubik et al., 2018). We found that, within the DNA contexts that we tested, Rap1 can modulate RSC activity and limit RSC-dependent encroachment of nucleosomes onto its binding sites. This allows Rap1 to bias the direction of RSC remodeling and to stabilize an open NDR. An attractive model for this observation is that Rap1 may act as a “backstop” for RSC activity (Figure 7). The positioning of the Rap1 binding sites relative to the nucleosome dyad might play an important role in determining remodeling direction, as sliding nucleosomes over Rap1 binding sites carries an energetic penalty. Indeed, this model is supported by nucleosome positioning data for *RPL30* and related yeast promoters (Figure S7M; Kubik et al., 2018). Moreover, RSC- and Rap1-bound remodeling intermediates may provide an explanation for the observation of MNase-sensitive fragile nucleosomes at Rap1-bound promoters (Brahma and Henikoff, 2019, Kubik et al., 2015). Finally, upon displacing promoter nucleosomes, Rap1 bound to free DNA results in the long residence times observed for specifically bound Rap1 *in vivo* (Lickwar et al., 2012). Together, our studies thus provide a mechanistic model (Figure 7) of how Rap1 accesses chromatin and establishes an active promoter conformation.

## STAR★Methods

### Key Resources Table

**Table:** 

REAGENT or RESOURCE	SOURCE	IDENTIFIER
**Bacterial and Virus Strains**		

*Escherichia coli* BL21(DE3)pLysS	Merck (Novagen)	Cat#69451-3
*Escherichia coli* DH5α	New England Biolabs	Cat#C2987H
*Escherichia coli* DH10MB	Schalch laboratory: (https://www.schalchlab.org/)	N/A
Sf9	Schalch laboratory	N/A

**Chemicals, Peptides, and Recombinant Proteins**		

SF-4 Baculo Express ICM	Bioconcept	Cat#9-00F38-I
Sf-900 II SFM	Life Technologies	Cat#10902096
FuGENE HD	Promega	Cat#E2311
Cre Recombinase	New England Biolabs	Cat#M0298
Bsa-I HF	New England Biolabs	Cat#R3535
DraIII-HF	New England Biolabs	Cat#R3510
ScaI-HF	New England Biolabs	Cat#R3122
EcoRV-HF	New England Biolabs	Cat#R3195
T4 DNA Ligase	New England Biolabs	Cat#M0202
Catalase from bovine liver	Sigma	Cat#C40
Glucose Oxidase from *Aspergillus niger*	Sigma	Cat#49180
Janelia Fluor-549 Halo	Lavis laboratory, Janelia	N/A
Alexa Fluor 568 NHS Ester	Life Technologies	Cat#A20003
Alexa Fluor 647 NHS Ester	Life Technologies	Cat#A20006
Cy3B NHS Ester	GE Healthcare	Cat#PA63101
Micrococcal nuclease	New England Biolabs	Cat#M0247S

**Critical Commercial Assays**		

MinElute PCR Purification Kit	QIAGEN	Cat#28004
TruSeq ChIP Sample Preparation Kit	Illumina	IP-202-1012
TruSeq ChIP Sample Preparation Kit 1024	Illumina	IP-202-1024

**Deposited Data**		

Full gel images	This paper	Mendeley data; https://doi.org/10.17632/btx2dbdg8h.1
Single-molecule datasets	This paper	https://zenodo.org (DOI: 10.5281/zenodo.3260205; 10.5281/zenodo.3270526; 10.5281/zenodo.3269823; 10.5281/zenodo.3270478; 10.5281/zenodo.3269904; 10.5281/zenodo.3269880
MNase-Seq datasets	This paper	GEO: GSE134143

**Experimental Models: Organisms/Strains**		

*Saccharomyces Cerevisiae* HHY168 RAP1(1-134)-FRB1-RAP1(136-827)-LEU2 (YJB26)	This paper	N/A

**Oligonucleotides**		

See Table S2.	IDT	N/A

**Software and Algorithms**		

ImageJ 1.50D	NIH	https://imagej.nih.gov/ij/
Origin 9.1G	OriginLab	https://www.originlab.com/
Nikon Elements 4.20.00	Nikon	https://www.nikon.com
Image Lab	Bio-rad	https://www.bio-rad.com
Custom MATLAB scripts (MATLAB R2016b)	Mathworks	N/A
Genomics analysis at Galaxy	-	https://usegalaxy.org

### Lead Contact and Materials Availability

Further information and requests for resources and reagents should be directed to and will be fulfilled by the Lead Contact, Beat Fierz (beat.fierz@epfl.ch). Plasmids and cell lines are available without restrictions upon request to the Lead Contact.

### Experimental Model and Subject Details

#### Cell lines and culture conditions

The *S. cerevisiae* strain HHY168 (carrying RAP1(1-134)-FRB1-RAP1(136-827)-LEU2 (YJB26)), containing indicated plasmids, was used for the experiments. Cells were grown in SC-His-Ura medium, containing 2% raffinose. For anchor-away experiments, cells were treated with rapamycin at 1 μg/ml, followed by growth in medium containing 2% galactose or 2% raffinose.

#### Cell lines for protein expression

Rap1 was expressed in Sf9 cells, cultured in suspension in sf900-II (GIBCO) medium. Histones were expressed in *E*. coli BL21 pLysS cells (Merck - Novagen) in LB medium.

#### Cell lines for DNA production

DNA constructs were expressed in *E*. coli DH5α (New England Biolabs) using 2xTY medium.

### Method Details

#### Expression and purification of Rap1-Halo

The Strep-MBP-TEV-Rap1-Halo construct (Figure S2F) was cloned into pACEBac1 (Geneva Biotech) and baculovirus particles were generated using the Geneva Biotech system per manufacturer’s instructions.

For Rap1 expression, 1L cultures of Sf9 cells were grown to 2 - 2.5x10^6^ cells/mL. Subsequently, the cells were infected with baculovirus, and the cultures were incubated for 3 days at 27°C, before harvesting through centrifugation (1500 r*cf.*, 4°C for 20 min). Supernatants were discarded and pellets were resuspended in PBS, containing protease inhibitors (Roche) (10 mL PBS/L of culture), flash frozen and kept at −80°C.

For a typical purification of Rap1-Halo, 12-15 g of frozen pellets were thawed at room temperature with 36 mL of lysis buffer (200 mM KCl, 2 mM DTT, 100 mM Tris-HCl (pH 7.5), 50 mM MgOAc, 0.1% NP-40, Protease inhibitor cocktail (Roche), 1mM PMSF and 20 μL DNaseI (NEB)). Pellets, were stirred with a magnetic stir bar until fully thawed and then kept on ice. The lysate was spun for 35 min at 35000 rpm at 4°C (Ti70 rotor, Beckman Coulter) and the supernatant was filtered through a 5 μm syringe filter (Millex, Millipore). The cleared lysate was loaded onto a Strep-Trap column (GE, AKTA system), pre-equilibrated with lysis buffer. The column was washed with storage buffer (200 mM KCl, 10 mM HEPES pH 7.6, 50 mM MgOAc, and 5 mM β-mercaptoethanol (βME)) and the protein was eluted with 5 x column volumes (CV) of elution buffer (storage buffer containing 2.5 mM desthiobiotin). Fractions containing Rap1 were identified by SDS-Page (Figures S2G and S2H), pooled and concentrated to ∼500 μL total volume using Amicon 10k molecular weight cut-off (MWCO) centrifugal filters. The protein concentration was determined using UV spectroscopy. The MBP tag was subsequently removed by TEV protease digestion at 4°C (Figure S2I). For labeling, Janelia Fluor-549 HaloTag (Janelia, JF-549) was added at a protein to dye ratio of 1:1.5 followed by incubation for 1h. Labeled Rap1 was finally purified by size exclusion chromatography (SEC) using a Superose6 10/300 GL column (GE healthcare) in storage buffer using a flow-rate of 0.4 mL/min (Figure S2J). Fractions were analyzed using SDS-PAGE (Figure S2K), clean fractions were pooled, concentrated (Amicon 10k MWCO filter) and protein concentrations were determined using UV spectrophotometry (at A280 and A571). Finally, labeling efficiency was calculated by using the extinction coefficients for Rap1 (107’065 mol^-1^ cm^-1^) and JF-549 (101’000 mol^-1^ cm^-1^). Typical labeling efficiency was found to be > 90%.

#### Expression and purification of recombinant histones

Histones were expressed and purified as described in Kilic et al. (2015). Briefly, individual *wild-type* human histones were cloned into pet15b plasmid vectors and expressed in BL21 DE3 plysS cells. Cells were grown in LB media containing 100 μg/mL ampicillin and 35 μg/mL chloramphenicol at 37°C until the OD_600_ reached 0.6. Expression was induced by IPTG addition to a final concentration of 0.5 mM. After 3 h expression, cells were harvested by centrifugation and resuspended in lysis buffer (20 mM Tris pH 7.5, 1 mM EDTA, 200 mM NaCl, 1 mM βMe, Roche protease inhibitor) and frozen. Cells were lysed by freeze-thawing and sonication. Inclusion bodies were harvested by centrifugation. The inclusion body pellet was washed once with 7.5 mL of lysis buffer containing 1% Triton and once without. Inclusion body pellets were resolubilized in resolubilization buffer (6 M GdmCl, 20 mM Tris pH 7.5, 1 mM EDTA, 1 mM βMe) and dialyzed into urea buffer (7 M urea, 10 mM Tris, 1 mM EDTA, 0.1 M NaCl, 5 mM 1 mM βMe, pH 7.5). Histones were purified by cation exchange chromatography using a HiTrap SP HP 5 mL column (GE Healthcare). Fractions were analyzed by SDS-PAGE and pooled, followed by dialysis into water and lyophilization. Final purification was performed by preparative RP-HPLC. Purified histones were lyophilized and stored at −20°C until used for octamer refolding.

#### Large scale generation of recombinant plasmids

Plasmids containing recombinant DNA fragments for chromatin DNA assembly, which have been prepared previously (Kilic et al., 2018b) (recP1, recP5) or were newly generated using restriction digestion and ligation of previous fragments (recP1P2 or recP4P5, Figure S4B) were transformed into DH5α cells (for sequence information see Table S1). Cells were cultured overnight in 6L 2xTY medium and harvested by centrifugation. For alkaline lysis, the cells were resuspended in 120 mL lysis solution I (50 mM glucose, 25 mM Tris pH 8, 10 mM EDTA). 240 mL lysis solution II (0.3 M NaOH, 1% SDS) was added and mixed by stirring. 240 mL lysis solution III (4 M KAc, 2 M acetic acid) was added to neutralize the solution which was left at 4°C for 15 min. After centrifugation, the supernatant was passed through Miracloth (Merck). Plasmid DNA was collected by isopropanol precipitation: 0.52 volume equivalents of isopropanol was added followed by centrifugation at 11’000 x g for 20 min at 4°C. The DNA pellet was dissolved in TE 10/50 (10 mM Tris pH 7.5, 50 mM EDTA) in the presence of 100 units of RNase A, and digested for 2 h at 37°C. To perform SEC the buffer was adjusted to 2M KCl (10 mM Tris, 50 mM EDTA and 2 M KCl). The plasmid was then purified in the same buffer on a XK 50/30 column (GE Healthcare) containing a bed of 550 mL Sepharose 6 Fast Flow (GE Healthcare). Eluted plasmid DNA from was precipitated with isopropanol. The pellet was finally dissolved in TE 10 / 0.1 (10 mM Tris pH 7.5, 0.1 mM EDTA) and stored at −20°C.

#### Large scale restriction digest and purification of recombinant plasmids

Purified plasmid DNA was collected by isopropanol precipitation and the DNA pellet was dissolved in milliQ H_2_O. For a typical reaction, either 200 units of DraIII-HF (NEB) (for recP1P2) or 200 units of BsaI-HF (NEB) (for recP4P5) or 200 units of both DraIII-HF and BsaI-HF (NEB) (for recP1 and recP5) were added to 200 pmol of plasmid DNA in 200μl 1x NEB CutSmart buffer. After 8-10 h digestion at 37°C, digestion progress was analyzed by gel electrophoresis on a 1% agarose gel (run in 1 x TBE running buffer, 100 V, for 50 min) to check completeness. If required, the digestion was pushed to completion by adding another 100 units of enzyme and incubation for further 8-10 h at 37°C. Once the digestion was complete, 100 units EcoRV-HF (NEB) was added and left 8-10 h at 37°C. Complete digestion was verified by electrophoresis as described above. If the digestion was not complete an additional 50 units of enzyme was added and left 8-10 h at 37°C. Once the digestion was complete, the desired chromatin DNA fragments were purified from the plasmid remnants through successive PEG precipitations. This involves adding 40% PEG 6000 to the digestion reactions until a final concentration of 5%–6% PEG 6000 was reached. Additionally, the NaCl concentration was adjusted to 0.5 M. The sample was then spun at 20’000 x g at 4°C for 20 min. The supernatant was collected, and PEG 6000 was added to the supernatant to increase the final PEG % by increments of 0.5%. The sample was then spun at 20’000 x g at 4°C for 20 min. This was repeated until a suitable purity was achieved. Finally, the chromatin DNA fragments were isolated using QIAquick PCR purification spin columns (QIAGEN).

#### Oligonucleotide labeling

Fluorescently labeled oligonucleotides were generated as described in Kilic et al. (2018b). Briefly, 5-10 nmol of single stranded oligonucleotide, containing amino modified C6 dT, was diluted in 25 μl 0.1 M sodium tetraborate, pH 8.5. 5 μl of a 5 mM stock of succinimidyl-ester modified fluorophore (Alexa 568, Alexa 647 or Cy3B) were added to the reaction mix and left shaking at room temperature for 4 – 8 hours. For a table enumerating all labeled oligonucleotides see Table S2.

Reaction progress was analyzed by RP-HPLC using a gradient from solvent A (95% 0.1M triethylammonium acetate (TEAA) pH 7, 5% ACN) to solvent B (70% 0.1M TEAA pH 7, 30% ACN) on a 3 μm 4.6x150 mm InertSustain C18 column (GL sciences) over 20 min. More dye was added when required. For purification, the labeled DNA was ethanol precipitated (by the addition of 2.75 equivalents of cold ethanol, 0.3M NaOAc pH 5.2, followed by centrifugation at 20’000 x g at 4°C for 20 min) twice successively to remove excess unconjugated dye. The DNA pellet was finally dissolved in 100 μl solvent A and purified by HPLC. The purified DNA was finally ethanol precipitated and dissolved in milliQ water to a concentration of 2.5 μM.

#### Production of labeled DNA fragments

Labeled DNA was prepared by PCR (fragments P2, P3_S1, P3_S2, P3_S2^∗^, P3_S1S2, P3_Rpl30, P3_Rpl30_S1 and P4, for sequences and labeling schemes, see Tables S1, S2, and S3). For a typical reaction, 96 × 50 μL PCR reactions in 1 x ThermoPol reaction buffer (NEB) were prepared using template (0.01 ng μL^-1^), forward primer (0.250 μM), reverse primer (0.250 μM), dNTPs (0.2 mM, NEB) and Taq DNA polymerase (1.25 units, NEB). A typical program included an initial step of 12 s at 94°C, followed by 30 cycles of 12 s at 94°C, 12 s annealing at 58-65°C and 12 s extension at 72°C. Final extension was also done at 72°C for 12 s. PCR reactions were subsequently purified using QIAquick PCR purification spin columns (QIAGEN).

About 0.33 nmol of PCR generated DNA (P3_S1, P3_S2, P3_S2^∗^, P3_S1S2, P3_Rpl30 and P3_Rpl30_S1, Table S1) was digested in 200 μl of 1 x CutSmart buffer using 100 units of BsaI-HF (NEB) and 100 units of DraIII-HF (NEB) for 8-10h at 37°C. The progress of the digestion was analyzed on a 2% agarose gel (running conditions: 1 x TBE, 110 V for 50 min). Finally the DNA fragments were purified using QIAquick PCR purification spin columns (QIAGEN) and the concentration was determined by UV spectroscopy.

#### Ligation and purification of 1 × 601 DNA to biotin anchor

For the generation of nucleosome DNA for single-molecule experiments, a biotin containing anchor (Anchor_rev, Table S2) was annealed to its complementary strand containing a phosphorylated 5′- BsaI overhang (P3_Anchor_fwd, Table S2) and a 10-fold excess was added to 150-300 pmol (∼20-40 μg) of digested PCR generated DNA (P3_S1, P3_S2, P3_S2^∗^ and P3_S1S2) in 100 μl 1x T4 ligase buffer (NEB). Upon complete ligation of digested DNA, excess biotin anchor was removed by PEG precipitation. Finally, the DNA fragments were purified using QIAquick PCR purification spin columns (QIAGEN) and the concentration was determined by UV spectroscopy.

#### Mononucleosome (MN) nucleosome formation

Nucleosomes (MN_S1, MN_S2, MN_S2^∗^, MN_S1S2, MN_S1_FRET, MN_S2_FRET, MN_Rpl30, MN_Rpl30_S1, MN_Rpl30_S1_FRET, Table S3) were prepared following Dyer et al. (2004). Typically, 1-5 μg of labeled and biotinylated DNA (P3_S1, P3_S2, P3_S2^∗^ and P3_S1S2) was combined with purified refolded octamers at experimentally determined ratios (1:1 to 1:2, DNA to histone octamer) in 10 μl TE (10 mM Tris-HCl pH 7.5, 1 mM EDTA) supplemented with 2 M KCl. After a 30 min incubation period at room temperature, 10 μl TE was added and further incubated for 1 h. This was followed by sequential addition of 5 μl TE, 5 μl TE and finally 70 μl TE with 1 h incubation periods in between each addition, to arrive to 0.2 M KCl. Samples were then spun at 20’000 x g for 10 min at 4°C and the supernatant was kept on ice. To determine the quality of MN assemblies, 0.8% Agarose 0.25 x TB gels were run at 90 V on ice for 90 min (Figures S2A–S2E, S5C, and S5K).

#### Electrophoretic mobility shift assays (EMSA)

EMSAs to determine Rap1 binding to DNA were done in single-molecule imaging buffer (IB, 50 mM HEPES pH 7.5, 130 mM KCl, 10% v/v glycerol, 0.005% v/v Tween 20, 2 mM Trolox, 3.2% w/v glucose), in the presence of 50 ng/μl poly-d(I-C) (Roche) and with 20 μL total volume. Typically, 200 nmol stocks of DNA and 3 μM stocks of Rap1-Halo were prepared and serially diluted to desired concentrations. Reactions were mixed by pipetting and left for 10 min at room temperature. Sucrose was added to a final concentration of 8% and reactions were loaded onto 5% Polyacrylamide gels run in 0.5 x TBE at 100 V for 60 min. Images were taken using ChemiDoc MP (Biorad) (Figures S1D and S1E). For densitometry quantifications, ImageLab (Biorad) software was used for band quantification of bound and unbound fraction of DNA. The data was analyzed in Origin (OriginLab) by non-linear curve-fitting using a sigmoidal function to determine K_d_.

#### Convergent 3-piece and 5-piece convergent DNA ligation for synthesis of 12x601 DNA

Singly-labeled and biotinylated 12x601 DNA was produced as shown in Figure S4A. Typically, 50-60 pmol of PEG purified restriction enzyme digested recP1P2 and an excess 1x601 P3 (P3_S1 and P3_S2) (between 20%–30% excess) were added to 200 μL 1 x T4 ligase buffer containing 400 units of T4 ligase. The reaction was followed using 1% agarose gels (Figure S4C). Upon completion, P1P2P3 was PEG purified (Figure S6F) and added to excess P4P5 and biotin labeled anchor (20%–30% excess P4P5 and 10-fold excess biotin anchor). The reaction was followed using 1% Agarose gels (Figure S4D). Upon completion, the complete DNA P1P2P3P4P5A was PEG purified and subsequently purified using Qiaquick PCR purification spin columns (QIAGEN), the concentration was determined by UV spectrophotometer (Figure S4E).

The FRET pair Cy3B and Alexa647, were site-specifically introduced respectively on P2 and P4 at the 39-base-pair position relative to the dyad in the 601 sequence (Figure S6A). About 30 pmol of each piece was used for 5-piece convergent DNA ligation to produce two intermediate 6 × 601 pieces as followed: recP1 was ligated to Cy3B-labeled P2 in 20% excess for 2 h using T4 DNA ligase, then unlabeled P3 in 20% excess relative to P2 was added and left to ligate another 15 h. Similarly, recP5 was ligated to 20% excess Alexa647-labeled P4 for 15 h (Figure S6D). Singly-labeled 6x601 intermediate fragments P1-3 and P4-5 were PEG purified from individual pieces. Pellets containing enriched fragments were collected, dissolved in 50 μL TE buffer (10 mM Tris, pH 8.0, 0.1 mM EDTA), and used for the final ligation (Figure S6E). A biotinylated anchor was added into the final ligation of 2 intermediate 6x601, and the reaction was proceeded for 15 h at room temperature. PEG precipitation was performed similarly to previous step, and the enriched final products were collected and purified using Qiaquick PCR purification spin columns (QIAGEN), the concentration was determined by UV spectrophotometer.

#### Reconstitution of 12-mer chromatin fibers

Chromatin fibers (CH_S1, CH_S2, CH_NS, CH_NS_FRET and CH_S2_FRET, Table S3) were reconstituted from singly/doubly-labeled and biotinylated 12x601 DNA and wild-type recombinantly purified human histone octamers. In a typical dialysis, 200-300 pM 12x601 DNA, 0.5-1 equivalents of MMTV DNA and reconstituted octamers (using experimentally determined DNA:octamer ratios) were added to a micro-dialysis unit (Thermo Scientific, Slide-A-Lyzer – 10’000 MWCO), then dialyzed in TE buffer (10 mM Tris pH 7.5, 0.1 mM EDTA pH 8.0) with a linear gradient from 2 M to 10 mM KCl for 16-18 h, and finally kept in TEK10 buffer (10 mM Tris pH 7.5, 0.1 mM EDTA pH 8.0, 10 mM KCl) for another 1 h. Chromatin assemblies were centrifuged at 21’000 x g for 10 min at 4°C, the supernatant was then transferred to a fresh tube. The concentration and volume of the chromatin assemblies was determined using UV spectrophotometer. Chromatin assembly quality was controlled by the appearance of MMTV nucleosomes and ScaI digestion of 12x assemblies. Digestion reactions were analyzed on a 0.8% agarose gel and 5% TBE polyacrylamide gel electrophoresis. All experiments were carried out at 4°C (Figures S4F–S4H, S6F, and S6G).

#### Preparation of microfluidic chambers for sm-FRET/TIRF experiments

Cleaning, silanization and PEGylation of coverslips and glass slides was done described previously in Kilic et al. (2015). Briefly, coverslips (24 × 40 mm, 1.5 mm thickness) and glass slides (76 × 26 mm with 2 rows of 4 holes drilled) were sonicated for 20 min in 10% Aconox, rinsed with milliQ water and the procedure repeated sequentially with acetone and ethanol. Both coverslips and glass slides were then placed in piranha etching solution (25% v/v 30% H_2_O_2_ and 75% v/v H_2_SO_4_) for minimum 2 h. After thorough washing with milliQ H_2_O, coverslips and slides were sonicated in acetone for 10 min, then incubated with 2% v/v aminopropyltriethylsilane (APTES) in acetone for 15 min, and dried. Flow-chambers were assembled from one glass slide and one coverslip separated by double-sided 0.12 mm tape (Grace Bio-labs) positioned between each hole in the glass slide, and the open ends were sealed with epoxy glue. Pipette tips were fitted in each of the 2 × 4 holes on each side of the silanized glass flow chambers as inlet reservoir and outlet sources and glued in place with epoxy glue. The glue was allowed to solidify for 30-40 min. Subsequently, 350 μL of 0.1 M tetraborate buffer at pH 8.5 was used to dissolve ∼1 mg of biotin-mPEG(5000 kDa)-SVA, and 350 μL from this was transferred to 20 mg mPEG (5000kDa)-SVA. This was centrifuged and mixed to homogeneity with a pipette before 40-45 μL aliquots were loaded into each of the four channels in the flow chamber. The PEGylation reaction was allowed to continue for the next 2½-4 h after which the solution was washed out with degassed ultra-pure water (Romil).

#### Single-molecule TIRF (sm-TIRF) co-localization microscopy measurements

Measurements were done according to Kilic et al. (2015). Objective-type smTIRF was performed using a fully automated Nikon Ti-E inverted fluorescence microscope, equipped with an ANDOR iXon EMCCD camera and a TIRF illuminator arm, controlled by NIS-elements and equipped with a CFI Apo TIRF 100x oil immersion objective (NA 1.49), resulting in a pixel size corresponding to 160 nm. Laser excitation was realized using a Coherent OBIS 640LX laser (640 nm, 40 mW) and coherent OBIS 532LS laser (532 nm, 50 mW) on a custom setup laser bench. Wavelength selection and power modulation was done using an acousto-optical tunable filter (AOTF) controlled by NIS-elements. Typical laser intensities in the objective used for measurements were 0.8 mW for both 532 nm and 640 nm laser lines. For all smTIRF experiments, flow channels were washed with 500 μL degassed ultrapure water (Romil), followed by 500 μL 1 x T50 (10 mM Tris pH 8, 50 mM NaCl) and background fluorescence was recorded with both 532 nm and 640 nm excitation. 50 μL of 0.2 mg/mL neutravidin was then injected and incubated for 5 min, and washed using 500 μL 1xT50. 50 pM of Alexa647 labeled DNA/mononuceosomes/12-mer chromatin assemblies were then flowed in for immobilization in T50 with 2 mg/mL bovine serum albumin (BSA, Carlroth) (25 × 50 μm imaging area was monitored using 640 nm excitation to check for sufficient coverage). 500 μL 1 x T50 was used to wash out unbound Alexa647 labeled DNA/mononuceosomes/12-mer chromatin assemblies. 50-100 pM JF-549 labeled Rap1-Halo (see table below for details) was flowed in using imaging buffer (50 mM HEPES pH 7.5, 130 mM KCl, 10% v/v glycerol, 0.005% v/v Tween 20, 2 mM Trolox, 3.2% w/v glucose, 1x glucose oxidase/catalase oxygen scavenging system and 2 mg/mL BSA). Images were recorded using the following parameters:

**Table:** 

	Camera t_on_ (msec)	Camera t_off_ (msec)	Orange channel # frames	Far-red channel: # frames	n repeat
DNA	100	600	1	1	5000
Mononucleosome	100	0.3	199	1	40
12-mer chromatin fiber	100	0.3	199	1	40

Here t_on_ denotes the camera integration time, whereas t_off_ indicates interspersed time intervals of camera inactivity.

Each experiment was repeated several times (see Table S4 for number of repeats), using at least two independently produced chromatin preparations on two different days.

#### Photobleaching test for JF-549 Rap1-Halo

Slides were prepared as described in the preceding sections. However, no BSA was added to imaging buffer (50 mM HEPES pH 7.5, 130 mM KCl, 10% v/v glycerol, 0.005% v/v Tween 20, 2 mM Trolox, 3.2% w/v glucose, 1x glucose oxidase/catalase oxygen scavenging system). JF-549 labeled Rap1-Halo was flown into the channel and nonspecifically adsorbed on the glass surface. Movies were recorded using continuous 532 nm illumination (t_on_ 50 msec and t_off_ 0.3 msec) using the indicated excitation laser powers (Figure S3A). Absolute laser power was determined using a laser power meter at the objective.

#### Ensemble FRET measurements

All measurements were performed using a Fluorolog®-3 Horiba Jobin Yvon spectrofluorometer, in T50 buffer (10 mM Tris pH 8, 50 mM NaCl) 60 μl total volume. Nucleosomes (final concentration of 25-30 nM) and Rap1 (0, 1, 2, 5, 10 equivalents) were mixed by pipetting in T50 buffer and left for 10 min room temperature to bind. Fluorescence emission spectra are taken from 585 nm to 700 nm (1 nm increments) using 578 nm as excitation wavelength. Spectra for DNA only, T50 only and donor only samples were taken. For a given sample, NaCl was added to 800 mM to observe nucleosome disassembly. FRET efficiency was calculated from donor emission:

$$E_{FRET}=1-\frac{F_{DA}}{F_{D}}$$

with *F*_*DA*_ denoting donor emission in the presence of acceptor, and *F*_*D*_ denoting donor emission in the donor-only sample. Additionally, reactions were loaded onto 0.5x TBE 5% polyacrylamide gels to check binding.

#### Single-molecule FRET (smFRET) measurements

Flow cell preparation and chromatin loading was performed as described in Kilic et al. (2018b) and the preceding paragraphs. Experiments were performed in FRET imaging buffer (40 mM KCl, 50 mM Tris pH 7.5, 2 mM Trolox, 2 mM nitrobenzyl alcohol (NBA), 2 mM cyclooctatetraene (COT), 10% glycerol and 3.2% glucose) supplemented with GODCAT (100x stock solution: 165 U/mL glucose oxidase, 2170 U/mL catalase). Experiments on chromatin remodeling effect of Rap1 were performed with imaging buffer containing 150 mM KCl, and 0.1 mg/mL of BSA was added to prevent nonspecific binding of Rap1 to glass surface. For Rap1 titration, unlabeled Rap1-Halo was used.

smFRET data acquisition was carried out with a micro-mirror TIRF system (MadCityLabs) using Coherent Obis Laser lines at 405 nm, 488 nm, 532 nm and 640 nm, a 100x NA 1.49 Nikon CFI Apochromat TIRF objective (Nikon) as well as an iXon Ultra EMCCD camera (Andor), operated by custom-made Labview (National Instruments) software.

For general smFRET imaging, a programmed sequence was employed to switch the field of view to a new area followed by adjusting the focus. The camera (at 500 EM gain) was triggered to acquire 1950 frames with 532 nm excitation and 100 ms time-resolution followed by a final change to 640 nm excitation.

Each experiment was repeated several times (see Tables S5 and S6 for number of repeats), using at least two independently produced chromatin preparations on two different days.

#### Nucleosome shift assays with RSC, Nap1 and Rap1

Purified RSC and recombinant yNap1 were used (for the purification, see Kurat et al., 2017). All reactions were performed in reaction buffer (10 mM Tris pH 7.4, 150 mM KCl, 3 mM MgCl_2_, 0.1 mg/mL BSA) and a total volume of 50 μl. The following components were added in sequential order MNs (to give a 20 nM final concentration), yNap1 (10 eq. yNap1: 1 eq. MNs), if required Rap1 (10 eq. Rap1: 1 eq. MNs), RSC complex (0.2 eq. RSC: 1 eq. MNs) and finally ATP (1mM). Reactions were placed at 30°C and 10 μl were taken for each time point, to which was added a 3-fold molar excess of plasmid DNA (compared to nucleosomes) containing a Rap1 binding site and returned to 30°C for 5 min. Reactions were then placed on ice until glucose was added to make 8% final concentration and loaded onto commercial Criterion Precast Gel (Biorad) 5% TBE, 1mm, run in 1x TBE at 200 V for 35-45 min on ice. Gels were stained in GelRed and imaged using ChemiDoc MP (Biorad) Figures S7A, S7B, and S7G). Leaving out Nap1 from the reaction did not affect RSC remodeling (Figure S7C). Remodeling assays using MNs containing fluorescently labeled octamers were also performed (Figure S7D) using the same conditions as described above. To model the RSC displaced nucleosome, an asymmetric PCR generated *P3_S12_remodelled* (Table S2) DNA fragment was used. This DNA was reconstituted into a nucleosome and incubated with Rap1 for 10 min at 30°C in reaction buffer (10 mM Tris pH 7.4, 150 mM KCl, 3 mM MgCl_2_, 0.1 mg/mL BSA), total volume of 10 μl. A 3-fold excess of plasmid DNA (compared to nucleosomes) containing a Rap1 binding site was added and returned to 30°C for 5 min. Reactions were then placed on ice until glucose was added to make 8% final concentration and loaded onto 5% polyacrylamide 0.5x TBE, 1.5 mm, run in 0.5x TBE at 120 V for 55-60 min on ice. Gels were stained in Gelred and imaged using ChemiDoc MP (Biorad) (Figure S7E). For the sequential remodeling experiment, nucleosomes were incubated with RSC and Nap1 for 90 min as described above. At 90 min, the RSC reaction was stopped by the addition of 30 mM EDTA pH 8.0. Then, Rap1 was added for 5 min at 30°C, followed by analysis on native PAGE (Figure S7F).

#### RSC sliding and MNase-seq

RSC sliding reactions were performed in reaction buffer (10 mM Tris pH7.4, 150 mM KCl, 3 mM MgCl_2_, 0.1 mg/mL BSA) and a total volume of 70 μl. The following components were added in sequential order MNs (to make 20nM final concentration), Nap1 (10 Nap1: 1 MN ratio), Rap1 (10 Rap1: 1 MN ratio, for w/o Rap1 MQ water was used as substitute), RSC complex (0.2 RSC: 1 MN ratio) and finally ATP (1 mM). Reactions were placed at 30°C for 90 min after which 10 μL was taken and glucose was added to make 8% final concentration and loaded onto commercial Criterion Precast Gel (Biorad) 5% acrylamide, 1mm, run in 1xTBE at 200 V for 35-45 min on ice. Gels were stained in Gelred and imaged using ChemiDoc MP (Biorad) (Figure S7G). To the remaining 60 μl, 60 μL 50mM Tris-HCl pH 8 and 10x NEB MNase buffer (M0247S) (to make final 1x) was added. This 120 μL total sample was split into 3 × 40 μL aliquots and to each either 6 units, 3 units or 1 unit of Mnase (M0247S) was added respectively and left to digest for 5 min at 37°C. To stop the reaction an equal volume of stop buffer was added (200 mM NaCl, 30 mM EDTA pH 8.0, 1% SDS) and left on ice for 5 min. Finally, 10 μg of Proteinase K (Sigma P2308) was added and left for 1h at 60°C and DNA fragments were isolated using QIAquick PCR purification spin columns (QIAGEN). For nucleosome only samples (t0), reactions were performed directly in 1x NEB Mnase buffer (M0247S), Mnase and Proteinase K digestion as well as DNA fragment purification was performed as described for RSC assay nucleosomes.

Following MNase digestion, DNA was purified using MinElute PCR Purification Kit (QIAGEN). The libraries were prepared using TruSeq ChIP Sample Preparation Kit (Illumina, Catalog IDs: IP-202-1012, IP-202-1024) according to manufacturer’s instructions. The libraries were sequenced on a HiSeq 4000 machine in 100 bp paired-end mode at the Genomics Platform of the University of Geneva (https://ige3.genomics.unige.ch/). Mapping of the sequencing data to the corresponding sequences was performed using Bowtie2 (sensitive end-to-end mode) on Galaxy (https://usegalaxy.org/). All densities were derived from read counts normalized to the total number of reads for each sample and BAM files was converted to bigWig files using bamCoverage and bigWig files converted to BedGraph format on Galaxy.

#### Yeast experiments

##### Plasmid construction

The pRS313-GALL plasmid was constructed by subcloning of *Sac*I and *Xba*I fragment from pRS416-GALL plasmid and inserted into pRS313 for construction of plasmid expressing RAP1 under the control of GALL promoter. The *RAP1* coding region was amplified using primers 5′-CATGTCTAGAATGTCTAGTCCAGATGATTTTGAAAC-3′ (Forward) and 5′-CATGCCCGGGTCATAACAGG TCCTTCTCAAAAAATC-3′ (Reverse) containing *Xba*I and SmaI sites and inserted into pRS313-GALL construct, digested with *Xba*I and SmaI. To construct pLR10-RPL30 plasmids, first RPL30 WT and RPL30-m1, RPL30-m2, and RPL30-m1/m2 mutants were cloned into pUC18 plasmid between *Sph*I and *Sac*I sites using primers 5′-ATGCGCATGCCTGCGTATATTGATTAATTGAA-3′ (Forward) and 5′-ATGCGAGC TCATATCATGCAGTACATTGACAGTATATCA-3′ (Reverse). Corresponding regions were then amplified by PCR using primers 5′-ATGCGTCGACATATCATGCAGTACATTGACAGTATATCA-3′ (Forward) and 5′-ATGC GCATGCCTGCGTATATTGATTAATTGAA-3′ (Reverse), and cloned into pLR10 plasmid just upstream of the YFP reporter gene at *Sph*I and SalI sites. The yeast RAP1 anchor away strain HHY168 *RAP1(1-134)-FRB1-RAP1(136-827)-LEU2* (YJB26) was co-transformed with the pRS415-GALL-RAP1 and pLR10-RPL30 plasmids.

#### *Cell lines and culture conditions*

The yeast cells, transformed with pRS313-GALL-RAP1 and pLR10-RPL30 plasmids, were grown overnight in SC-His-Ura containing 2% raffinose. Overnight cultures were diluted to OD600 0.1, grown at 30°C to OD600 0.3-0.4, and then treated with either vehicle (90% ethanol/10% Tween) or, for anchor-away, with rapamycin (1 mg/mL of 90% ethanol/10% Tween stock solution) at a final concentration of 1 μg/ml (Haruki et al., 2008) (1 μg/mL) for 1 hr to deplete FRB-tagged RAP1 protein. Following the rapamycin treatment, the strains were grown in medium containing 2% galactose for 2 hr to induce expression of RAP1 or 2% raffinose.

##### MNase digestion and nucleosome mapping

MNase digestion was performed as described (Kubik et al., 2015). Briefly, yeast cells were grown at 30°C for o/n in SC-His-Ura media containing 2% raffinose to OD_600_ 0.3-0.4, crosslinked with 1% formaldehyde for 5 min and quenched by the addition of 125 mM glycine for 5 min at room temperature. The cell pellets were resuspended in spheroplasting buffer (1 M sorbitol, 1 mM β-mercaptoethanol, 10 mg/mL zymolyase) after harvesting and incubated for 8 min at room temperature. Spheroplasts were washed twice using 1 mL of 1 M sorbitol and treated with different concentrations of MNase, ranging from 0.05 to 1.0 units. The samples were incubated at 37°C for 45 min in MNase digestion buffer (1M Sorbitol, 50 mM NaCl, 10 mM Tris pH 7.4, 5 mM MgCl_2_, 1 mM CaCl_2_, 1mM β-mercaptoethanol, 0.5 mM spermidine and 0.075% NP-40). Digestion reactions were stopped by the addition of EDTA (30 mM), the crosslinks were reversed with SDS (0.5%) and proteinase K (0.5 mg/mL) and incubated at 37°C for 1 h and then transferred to 65°C for at least 2 h. The DNA was isolated by phenol/chloroform/isoamyl alcohol (25:24:1) extraction, concentrated with ethanol and treated with RNase at 37°C for 1 h for monitoring on agarose gel (2%). MNase profiles were determined by qPCR of chromatin samples (previously digested with 0.5 units MNase) using a set of nested primer pairs covering the *RPL30* promoter region ∼561 bp upstream of the ATG.

##### Flow cytometry

Flow cytometry analysis was performed to detect the expression of a YFP reporter driven by RPL30 promoter and its variants in different conditions. Yeast transformants were grown to stationary phase overnight in appropriate media, the cells were diluted to OD600 0.1 the next day and grown to exponential phase at OD600 0.3-0.4. Upon flow cytometry, the cells were diluted 10-fold into SC-His-Ura media and immediately processed on Beckman Coulter Gallios Flow Cytometer. YFP-expressing cells were sorted by fluorescence-activated cell sorting (FACS) analyses using excitation lasers at 488 nm, and filtering emissions at 525 nm.

### Quantification and Statistical Analysis

#### Image processing, single-molecule trace extraction and trace analysis

Single-molecule trace extraction and trace analysis were done according to Kilic et al. (2015) with some adjustments. First, a background subtraction was performed for all Rap1-Halo binding movies using a rolling ball background subtraction in ImageJ (using 50 pixel rolling ball size). Using a custom built MATLAB (Mathworks) program suite, DNA/nucleosome or chromatin positions were detected via a local maxima approach. Sequential images were aligned using the far-red channel to compensate for stage drift. Fluorescence intensities (in the orange channel) were extracted from the stack within a 2 pixel radius of the identified DNA peaks. Every detected spot in the orange channel was fitted with a 2D-Gaussian function to determine co-localization with immobilized DNA/chromatin. Peaks exceeding an experimentally determined PSF width for a single JF-549 molecule were excluded from further analysis. Extracted fluorescence traces were filtered using a forward-backward non-linear filter (Chung and Kennedy, 1991) to reduce noise.

Residence times were determined using a semi-automatic procedure. Individual binding events were detected using a thresholding algorithm. Overlapping multiple binding events were excluded from the analysis. For each movie cumulative histograms were constructed from detect bright times (*t*_*bright*_) corresponding to bound Rap1 molecules, usually including data from ∼100 individual traces. The cumulative histograms from traces corresponding to individual DNA / MN / chromatin fibers were fitted with either di- or tri-exponential functions:

$$y=\sum_{i=1}^{2}A_{i}\;\exp\left(-t/\tau_{off,\;i}\right)\;\text{or}\;y=\sum_{i=0}^{2}A_{i}\;\exp\left(-t/\tau_{off,\;i}\right)$$

yielding nonspecific residence times τ_off,0_ or the specific residence times τ_off,1_ and τ_off,2_.

Cumulative histograms constructed from dark times (*t*_*dark*_), in between binding events, were fitted with mono-exponential functions:

$$y=A\;\exp\left(-t/k_{on,app}\right)$$

to obtain apparent on-rates. The detected on-rates contain both contributions from nonspecific and specific binding events. To calculate specific on-rates (*k*_*on*_), the contributions from nonspecific events have to be filtered out. To this end, measured *k*_*on*,app_ values were corrected using the amplitude contributions of nonspecific (*A*_*0*_) and specific binding events (*A*_*1*_*, A*_*2*_).

$$k_{on,specific}=k_{on}\left(\sum_{i=1}^{2}A_{i}/\sum_{i=0}^{2}A_{i}\right)$$

#### Single-molecule FRET (smFRET) conformation analysis

##### Calibration

Before each experiment, instrument calibration was performed by imaging 100-nm biotinylated Gold nanoparticles (Cytodiagnostics) with 532 nm excitation and 100 ms time-resolution over 10 s. Acquired calibration movies were analyzed using a custom-written Macro ImageJ to determine the signal-to-noise ratio (SNR) as follows:

$$SNR=\frac{P-B}{\sqrt{\sigma_{P}^{2}+\sigma_{B}^{2}}}$$

Where P and $\sigma_{P}$ are average and standard deviation of peak, and B and $\sigma_{B}$ are that of its background. Our standard calibration was performed with 12 mW of 532 nm excitation at 500 EM gain of the camera resulting in average SNR of 6.5-8.5. Moreover, at least well-separated 10 nanoparticles representing the field of view and appearing in both the donor and the acceptor channels were selected to generate a transformation matrix, which was further applied for aligning non-isotropically donor and acceptor images.

##### smFRET data analysis

FRET reporting on chromatin conformation as a function of ionic strength or Rap1 binding was recorded as described above.

For FRET calculation, the orange and far-red channel detection efficiency ratio γ and donor dye bleed-through parameter β were independently determined using double-stranded DNA oligonucleotides, where X and Y indicate respectively 5′-Amino-C6 and 5-C6-Amino-dT and labeled respectively with Cy3B and Alexa Fluor 647

X – 5′ – TAAATCTAAAGTAACATAAGGTAACATAACGTAAGCTCATTCGCG – Biotin
3′ – ATTTAGATTTCATTGTAYTCCATTGTATTGCATTCGAGTAAGCGC

For all recorded movies, background correction was performed in ImageJ using a rolling ball algorithm. Single-molecule kinetic trace extraction and analysis was performed in custom-written MATLAB software. Donor and acceptor channels were non-isotropically aligned using the nanoparticle based transformation matrix. Individual molecules were automatically detected in the initial acceptor image prior to donor excitation, and the same peaks were selected in the donor channel. Peaks that are (*i*) tightly clustered or (*ii*) above an intensity threshold of 8000 in the donor channel and 5000 in the acceptor channels indicating aggregation or (*iii*) do not appear in both donor and acceptor channels were excluded from analysis. Kinetic donor and acceptor fluorescence traces were extracted for each single-molecule. Selection criteria were similar to Kilic et al. (2018b). Traces were included if they exhibited: (*i*) a single bleaching event, (*ii*) constant total fluorescence emission > 2000 counts from combined donor and γ-corrected acceptor channel (*iii*) a constant baseline lasting for at least 2 s after donor bleaching, (i*v*) donor emission for at least 5 s and finally (*v*) the presence of acceptor dye. The last condition is verified as follows: If the donor dye bleaches first, acceptor emission must be detectable at the end of the experiment upon direct acceptor excitation. If the acceptor dye bleaches first, a significant increase is seen in the donor channel. From selected traces, donor $\left(F_{D}\right)$ and acceptor $\left(F_{A}\right)$ fluorescence emission intensity, FRET efficiency $\left(E_{FRET}\right)$ was calculated as follows:

$$E_{FRET}=\frac{F_{A}-\beta F_{D}}{F_{A}-\beta F_{D}+\gamma F_{D}}\;\;where\;\beta =\frac{F_{A,\;bleach}}{F_{D,\;bleach}}and\;\gamma =\frac{\Delta F_{A,\;bleach}}{\Delta F_{D,\;bleach}}$$

We determined the detection efficiency γ=0.423 and the bleed-through β=0.073 for the FRET pair Cy3B/Alexa647 with our experimental setup. These values were used to calculate $E_{FRET}$ for the selected traces, and construct $E_{FRET}$ histograms with a bin size of 0.02. $E_{FRET}$ histograms of each trace of length > 5 s were normalized to total counts. Final histograms of each independent measurement were fitted using 3 Gaussian functions as follows:

$$\sum_{i}A_{i}e^{-\frac{{\left(x-c_{i}\right)}^{2}}{2\sigma_{i}^{2}}}$$

Where $A_{i}$ is the amplitude or the height of the fitting peak, $c_{i}$ is the position of the center of the peak, and $\sigma_{i}$ is the standard deviation which controls the width of the Gaussian peak. The integral area of each peak was calculated as follows:

$$\int_{-\infty }^{\infty }A_{i}e^{-\frac{{\left(x-c_{i}\right)}^{2}}{2\sigma_{i}^{2}}}dx=A_{i}\sigma_{i}\sqrt{2\pi }$$

Where indicated, low-FRET (LF), medium-FRET (MF) and high-FRET (HF) refer respectively to the center of the Gaussian peak limited with $c_{i}<$ 0.2, 0.2 $\leq c_{i\;}\leq$ 0.4, and $c_{i}>$ 0.4. The percentage of LF-population at compaction conditions, i.e., in high salt or presence of Mg^2+^, indicates the fraction of uncompacted chromatin, and hence reports chromatin assembly quality. Control of chromatin compaction was performed, and only measurements on chromatin preparation giving $E_{FRET}$ histograms with < 50% of LF-population were selected for further analysis (Figure S6).

Dynamic traces were identified by fluorescence cross-correlation analysis, performed using the following function:

$$C_{D-A}\left(t\right)=\frac{\Delta F_{D}\left(0\right).\Delta F_{A}\left(t\right)}{\Delta F_{D}\left(0\right).\Delta F_{A}\left(0\right)}$$

Where $\Delta F_{D}$ and $\Delta F_{A}$ denote the variances of donor and acceptor fluorescence at time 0 or t. Only traces lasting for more than 10 s, and spending > 20% of the duration time at $E_{FRET}>$ 0.2 were included and fitted with a bi-exponential function. A dynamic trace is defined as the one showing a cross-correlation amplitude inferior to −0.1 and a relaxation time superior to 100 ms.

All Gaussian fit parameters and cross-correlation analysis are shown in Tables S5 and S6.

#### Statistical analysis

All results are presented as means with their standard deviation, unless otherwise indicated. Pairs of experimental values were compared using two-sided, homoscedactic Student’s t tests with a confidentiality interval of 5%: a p value below 0.05 was considered as statistically significant.

### Data and Code Availability

Microscopy data, evaluation scripts and detailed plasmid maps of expression vectors are available upon request. Full gels and WB images are deposited on Mendeley data (https://doi.org/10.17632/btx2dbdg8h.1). All single-molecule data is available from https://zenodo.org (https://doi.org/10.5281/zenodo.3260205; https://doi.org/10.5281/zenodo.3270526; https://doi.org/10.5281/zenodo.3269823; https://doi.org/10.5281/zenodo.3270478; https://doi.org/10.5281/zenodo.3269904; https://doi.org/10.5281/zenodo.3269880). All sequencing data were submitted to the GEO database as Series GSE134143.

## Supplemental Information

Document S1. Figures S1–S7 and Tables S1–S7

Document S2. Article plus Supplemental Information
