# Supplementary material for: Chromatin Fiber Invasion and Nucleosome Displacement by the Rap1 Transcription Factor
Source: Mol Cell. 2020 Feb 6;77(3):488–500.e9. doi: 10.1016/j.molcel.2019.10.025 (PMC7005674; doi:10.1016/j.molcel.2019.10.025)
Supplement: Document S1. Figures S1–S7 and Tables S1–S7 [file mmc1.pdf]

Molecular Cell, Volume 77

Supplemental Information

Chromatin Fiber Invasion and Nucleosome Displacement by the Rap1 Transcription Factor

Maxime Mivelaz, Anne-Marinette Cao, Slawomir Kubik, Sevil Zencir, Ruud Hovius, Iuliia Boichenko, Anna Maria Stachowicz, Christoph F. Kurat, David Shore, and Beat Fierz

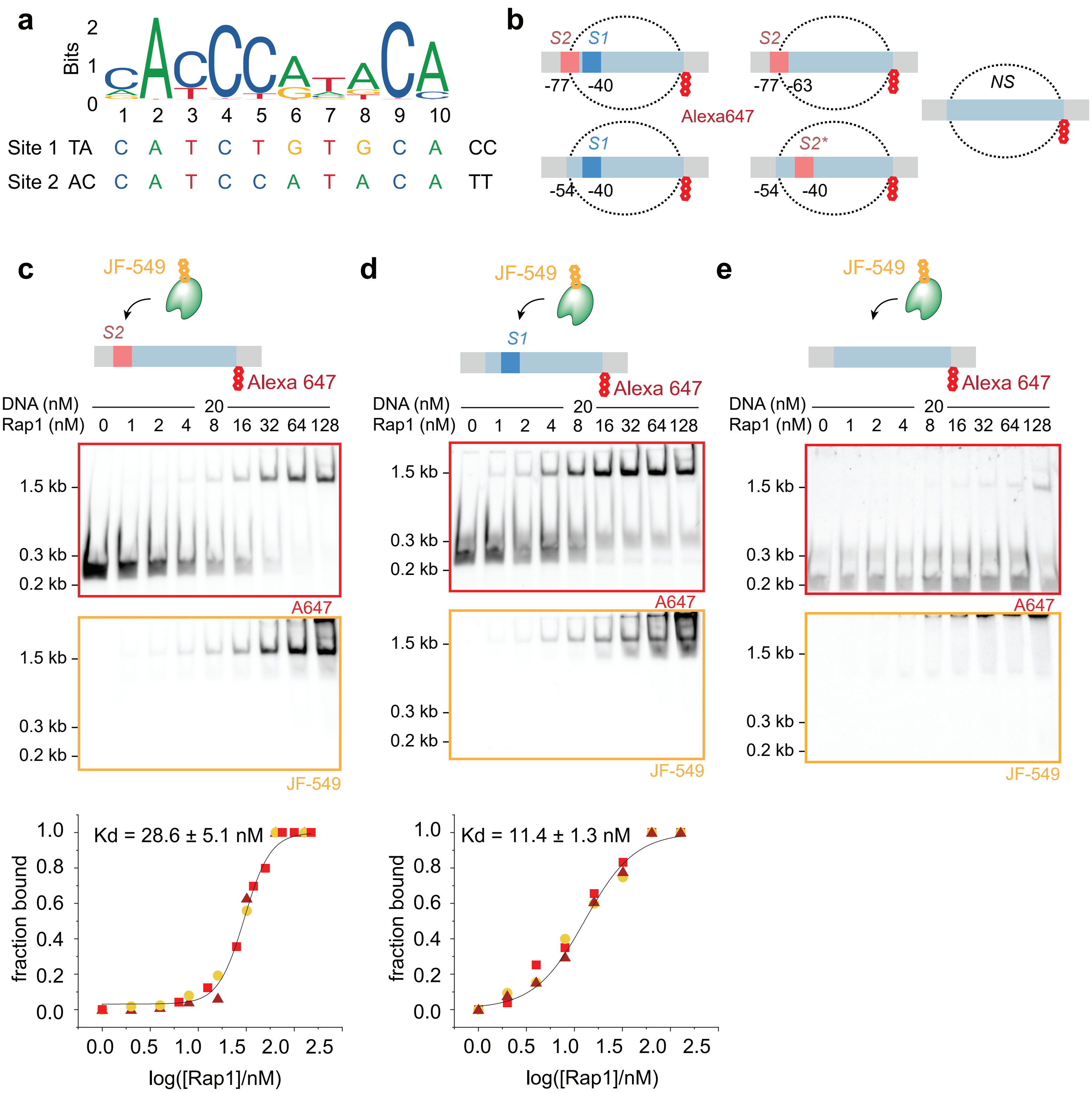

a
Bits
Site 1 TA C A T C T G T G C A CC
Site 2 AC C A T C C A T A C A TT
b
S2 S1
-77 -40
S2
-77 -63
NS
Alexa647
S1
-54 -40
S2*
-54 -40
c
JF-549
S2
Alexa 647
DNA (nM) 20
Rap1 (nM) 0 1 2 4 8 16 32 64 128
1.5 kb
0.3 kb
0.2 kb
A647
JF-549
Kd = 28.6 ± 5.1 nM
fraction bound
log([Rap1]/nM)
d
JF-549
S1
Alexa 647
DNA (nM) 20
Rap1 (nM) 0 1 2 4 8 16 32 64 128
A647
JF-549
Kd = 11.4 ± 1.3 nM
fraction bound
log([Rap1]/nM)
e
JF-549
Alexa 647
DNA (nM) 20
Rap1 (nM) 0 1 2 4 8 16 32 64 128
A647
JF-549

FIGURE S1

Figure S1. Related to Figure 1 | Mononucleosome design:

a) Rap1 consensus sequence. Aligned below are the sequences of the *RPL30* promotor Site 1 (*S1*) and Site 2 (*S2*).

b) Schematic representation of the DNA constructs used (see **Table S1-3** for sequence information); indicated are the position of the dye and the positions of the different binding sites relative to the dyad, additionally the position of the octamer is depicted by the dotted black line. Linker regions are shown in grey.

c-e) Electrophoretic mobility shift assays (EMSA) using labelled JF-549 Rap1-Halo and labelled DNA constructs.

c) Rap1 binding to DNA containing **P3_*S2***.

d) Rap1 binding to DNA containing **P3_*S1***.

e) Rap1 binding to DNA without Rap1 binding site. Titration of Rap1-Halo from 0 – 128 nM and incubated 10 min before loading on native-PAGE. DNA - Rap1-Halo complexes migrate to 1.5 kb and can be seen in both 647 and 549 illumination channels.

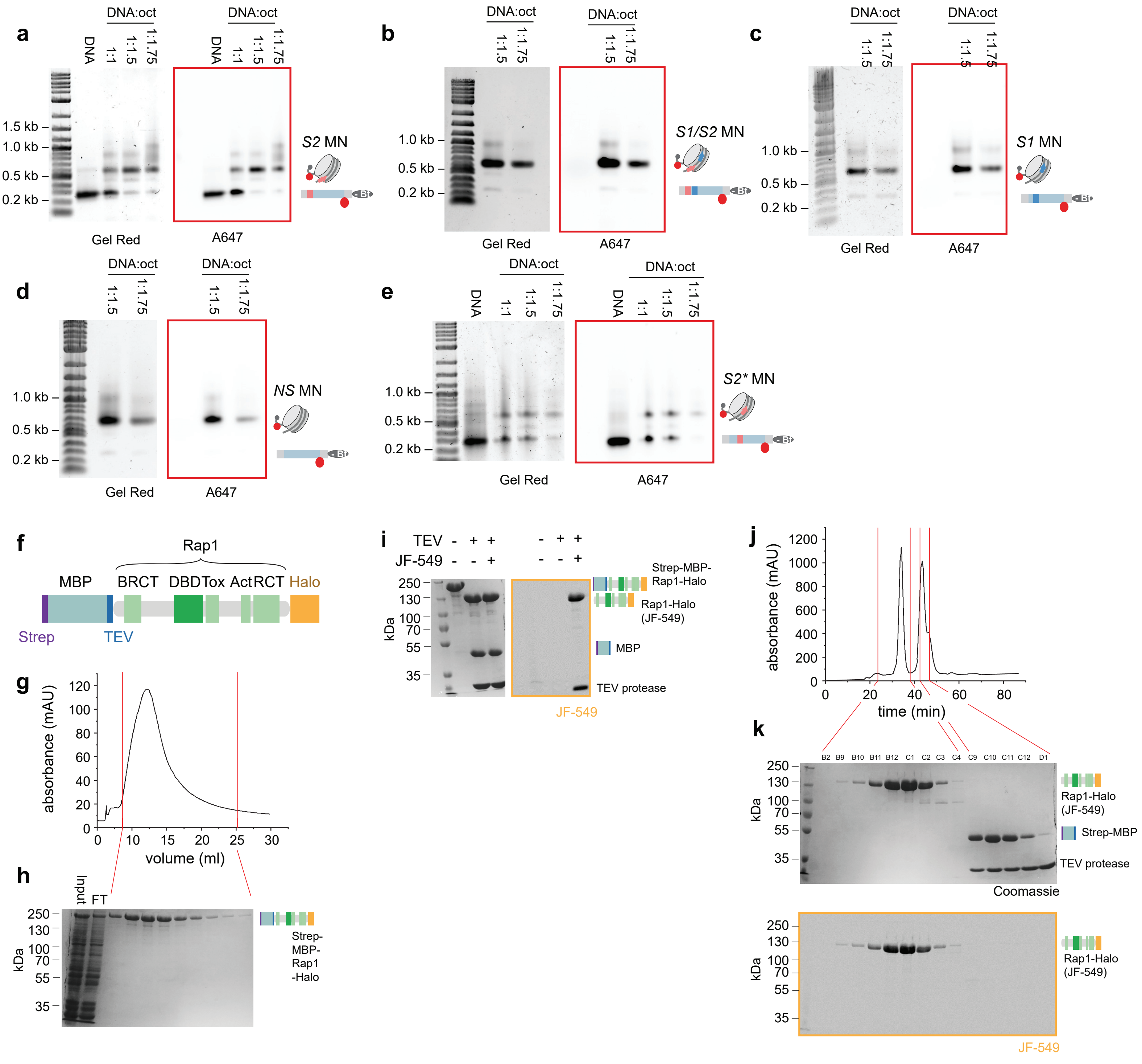
a
DNA:oct
DNA
1:1
1:1.5
1:1.75
1.5 kb
1.0 kb
0.5 kb
0.2 kb
Gel Red
A647
S2 MN
b
1.0 kb
0.5 kb
0.2 kb
S1/S2 MN
c
S1 MN
d
NS MN
e
S2* MN
f
Rap1
MBP
BRCT
DBD
Tox
Act
RCT
Halo
Strep
TEV
g
absorbance (mAU)
volume (ml)
h
Input
FT
kDa
Strep-MBP-Rap1-Halo
i
TEV
JF-549
Strep-MBP-Rap1-Halo
Rap1-Halo (JF-549)
MBP
TEV protease
JF-549
j
absorbance (mAU)
time (min)
k
B2
B9
B10
B11
B12
C1
C2
C3
C4
C9
C10
C11
C12
D1
Rap1-Halo (JF-549)
Strep-MBP
TEV protease
Coomassie
JF-549

FIGURE S2

Figure S2. Related to Figure 2 | Mononucleosome formation and Rap1 production:

a) MNs are reconstituted using **P3_*S2*** DNA with a biotin anchor at the indicated DNA to histone octamer ratio, and analyzed by agarose gel electrophoresis.

b) MNs containing **P3_*S1S2*** DNA with a biotin anchor.

c) MNs containing **P3_*S1*** DNA with a biotin anchor.

d) MNs containing **P3** DNA with a biotin anchor.

e) MNs containing **P3_*S2**** DNA with a biotin anchor.

f) Schematic representation of full-length baculovirus expressed Rap1-Halo construct. Strep tag (purple), Maltose binding protein (MBP, cyan), TEV cleavage site (blue), BRCA 1 C Terminus (BRCT) (green), DNA Binding Domain (DBD) (dark green), Toxicity region (Tox), Transcription Activation domain (Act), Rap1 C-Terminus (RCT) and Halo-Tag (orange).

g-h) Affinity chromatography profile and SDS-PAGE of corresponding fractions, including supernatant after centrifugation of lysed cells (input) and flow through (FT).

i) SDS-PAGE of TEV protease digestion and Halo-tag labelling using JF-549.

j-k) Gel filtration profile and SDS-PAGE of corresponding fractions.

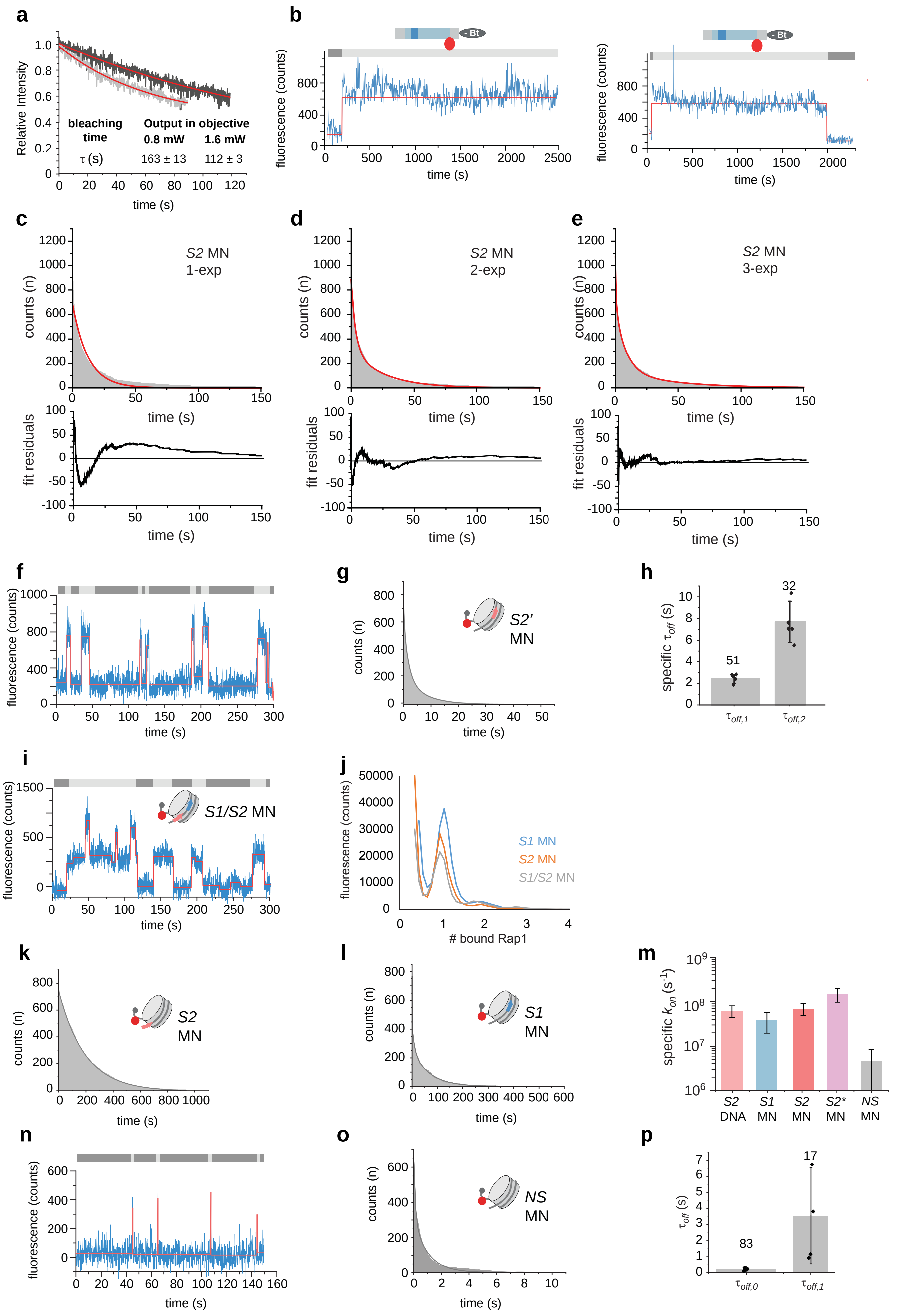
a
Relative Intensity
bleaching time
Output in objective
0.8 mW
1.6 mW
τ (s)
163 ± 13
112 ± 3
time (s)
b
Bt
fluorescence (counts)
time (s)
c
S2 MN
1-exp
counts (n)
time (s)
fit residuals
d
S2 MN
2-exp
e
S2 MN
3-exp
f
fluorescence (counts)
time (s)
g
S2' MN
counts (n)
time (s)
h
specific τ_off (s)
51
32
τ_off,1
τ_off,2
i
S1/S2 MN
fluorescence (counts)
time (s)
j
fluorescence (counts)
S1 MN
S2 MN
S1/S2 MN
bound Rap1
k
S2 MN
counts (n)
time (s)
l
S1 MN
m
specific k_on (s^-1)
S2 DNA
S1 MN
S2 MN
S2* MN
NS MN
n
fluorescence (counts)
time (s)
o
NS MN
counts (n)
time (s)
p
τ_off (s)
83
17
τ_off,0
τ_off,1

FIGURE S3

Figure S3. Related to Figure 2 | Mononucleosome binding and photobleaching analysis:

a) Immobilized Rap1-JF-549 was continuously illuminated at different power outputs (measured at objective). Intensity decays due to photobleaching were fitted with a single-exponential function yielding the bleaching kinetics of JF-549.

b) Example traces of biotinylated **P3_*S1*** DNA acquired using smTIRF (blue), with corresponding fit (red). Imagining was done at 0.75 frames/s (with respect to each laser line) to limit photobleaching, revealing very long binding events.

c) Example of a cumulative histogram of Rap1 residence times on a **P3_*S2*** mononucleosome (*S2* MN), fit with mono-exponential decay with residuals (below).

d) Same as in **c**) but fit with bi-exponential decay with residuals (below).

e) Same as in **c**) but fit with tri-exponential decay with residuals (below).

f) Example traces of biotinylated **P3_*S2**** DNA in reconstituted MNs, acquired using smTIRF (blue), with corresponding fit (red).

g) Example of a cumulative histogram and tri-exponential fit of MNs using **P3_*S2**** DNA (*S2** MN). Data from 100-200 traces.

h) Specific residence times of Rap1 binding to *S2** MN. The width of the bar indicates the relative population associated to each time constant.

i) Example trace of MNs containing **P3_*S1S2*** (*S1/S2* MN) acquired using smTIRF (blue), with corresponding fit (red), demonstrating a superposition of *S1* and *S2* binding kinetics.

j) Intensity distribution histograms for MNs containing either *S1*, *S2* or *S1S2*, reporting on the number of simultaneously bound Rap1 molecules (from 100 traces each). Under the measurement conditions (50 pM Rap1), even when two Rap1 binding sites are present, mostly single-binding events are observed.

k) Example of a cumulative histogram of dark times (t_{dark}) and mono-exponential fit of MNs containing **P3_*S2*** DNA (*S2* MN). Data from 200-300 traces.

l) Example of a cumulative histogram of t_{dark} and mono-exponential fit of MNs containing **P3_*S1*** DNA (*S1* MN). Data from 100-200 traces.

m) Specific association kinetics (k_{on}) of Rap1 to indicated MNs.

n) Example trace of biotinylated **P3** 601 DNA (*NS* MN) acquired using smTIRF (blue), with corresponding fit (red).

o) Example of a cumulative histogram and bi-exponential fit of *NS* MN. Data from 100-200 traces.

p) Residence times of Rap1 binding to *NS* MN. The width of the bar indicates the relative population associated to each time constant.

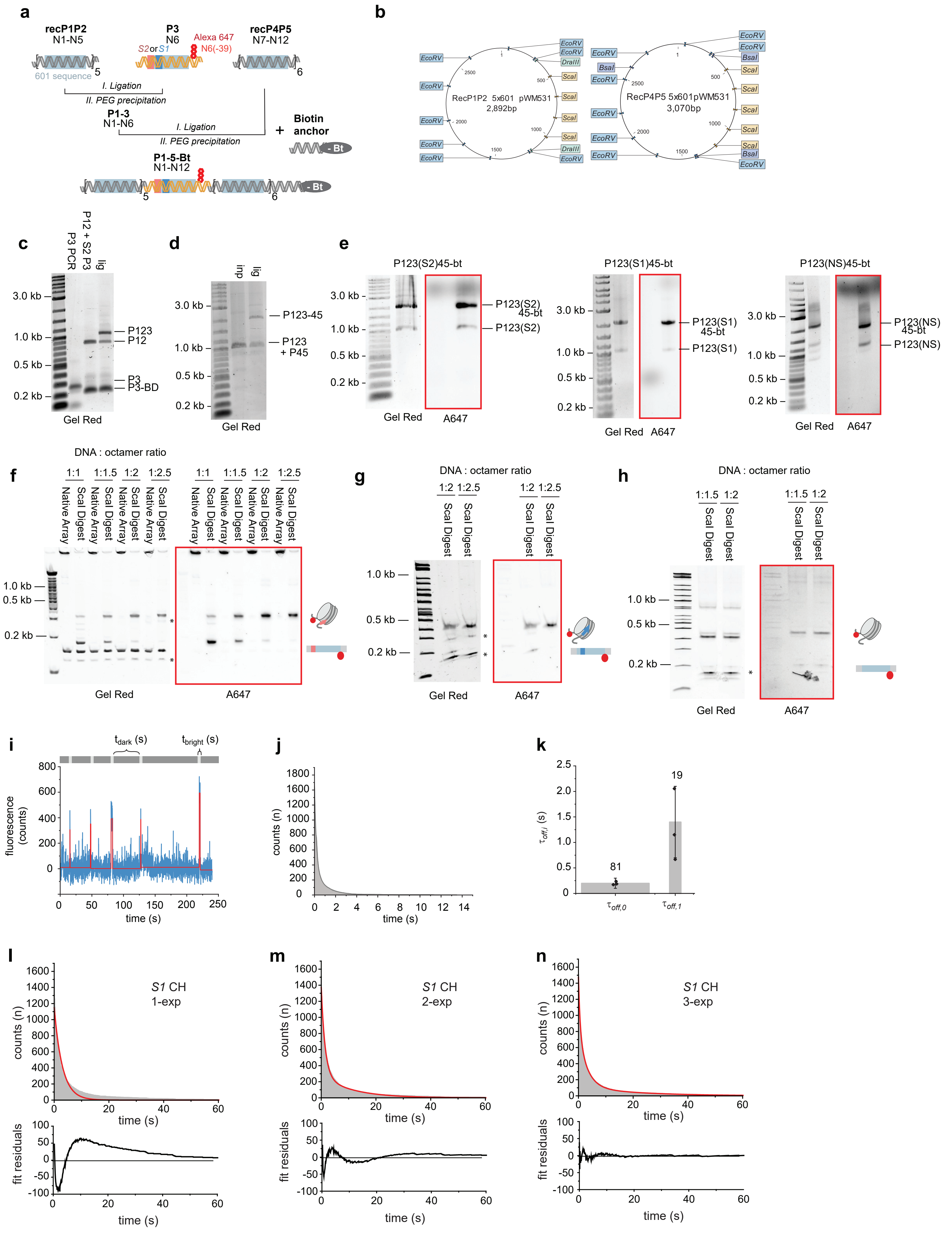

a
recP1P2
N1-N5
601 sequence
P3
N6
S2 or S1
Alexa 647
N6(-39)
recP4P5
N7-N12
I. Ligation
II. PEG precipitation
P1-3
N1-N6
Biotin anchor
P1-5-Bt
N1-N12
- Bt
b
RecP1P2 5x601 pWM531
2,892bp
RecP4P5 5x601pWM531
3,070bp
EcoRV
ScaI
DraIII
BsaI
c
P3 PCR
P12 + S2 P3
lig
P123
P12
P3
P3-BD
Gel Red
d
inp
lig
P123-45
P123 + P45
Gel Red
e
P123(S2)45-bt
P123(S1)45-bt
P123(NS)45-bt
P123(S2) 45-bt
P123(S2)
P123(S1) 45-bt
P123(S1)
P123(NS) 45-bt
P123(NS)
Gel Red
A647
f
DNA : octamer ratio
1:1
1:1.5
1:2
1:2.5
Native Array
ScaI Digest
Gel Red
A647
g
DNA : octamer ratio
1:2
1:2.5
ScaI Digest
Gel Red
A647
h
DNA : octamer ratio
1:1.5
1:2
ScaI Digest
Gel Red
A647
i
t_{dark} (s)
t_{bright} (s)
fluorescence (counts)
time (s)
j
counts (n)
time (s)
k
$\tau_{off,i}$ (s)
81
19
$\tau_{off,0}$
$\tau_{off,1}$
l
S1 CH
1-exp
counts (n)
time (s)
fit residuals
m
S1 CH
2-exp
counts (n)
time (s)
fit residuals
n
S1 CH
3-exp
counts (n)
time (s)
fit residuals

FIGURE S4

Figure S4. Related to Figure 3| Chromatin assembly:

a) Schematic representation of the ligation scheme used to generate 12-mer chromatin arrays with 3-piece ligation. Convergent ligation from sequential ligations and PEG purifications of individual pieces.

b) Scheme of recombinantly expressed rec**P1P2** and rec**P4P5** pieces containing 5x601 and 6x601 sequences respectively. These plasmids are engineered to simplify PEG precipitation purification (*EcoRV*) and nucleosome quality control (*ScaI*).

c) Ligation of individually purified rec**P1P2** and **P3** (**P3_*S1***, **P3_*S2*** and **P3**) pieces, using excess **P3**. **d**) Ligation of **P1P2P3** with **P4P5-bt**.

e) Pooled **P1P2P3P4P5-bt** fractions after PEG purification for **P123_*S2*-45-bt (CH_*S2*)**, **P123_*S1*-45-bt (CH_*S1*)** and **P12345-bt (CH_*NS*)** 12-mer DNA construct.

f) Analysis of chromatin formation using **CH_*S2*** DNA by *ScaI* restriction digestion. Different DNA to octamer ratios were tested to find optimal 601 array saturation conditions. The asterisks indicate free MMTV buffer DNA and MMTV buffer nucleosomes. We see array saturation at 1:2.5 DNA to octamer ratio.

g) Gel of **CH_*S1*** Array assembly showing saturation at 1:2.5 DNA to octamer ratio.

h) Gel of 12-mer **CH_*NS*** Array assembly showing saturation at 1:2 DNA to octamer ratio.

i) Example trace from smTIRF experiments using 12-mer **CH *NS*** chromatin without Rap1 site.

j) Example cumulative histogram of 12-mer **CH *NS*** chromatin smTIRF measurements. The data fit by a bi-exponential decay function.

k) Residence times for Rap1 on 12-mer **CH *NS*** chromatin showing $\tau_{off,1}$ 0.2 ± 0.1 s and $\tau_{off,2}$ 1.4 ± 0.7 s. The width of the bars indicate the percentage of events associated with the indicated time constants.

l) Example of a cumulative histogram of Rap1 residence times on a **CH_*S1*** chromatin fiber (*S1* CH), fit with mono-exponential decay with residuals (below).

m) Same as in **l**) but fit with bi-exponential decay with residuals (below).

n) Same as in **l**) but fit with tri-exponential decay with residuals (below).

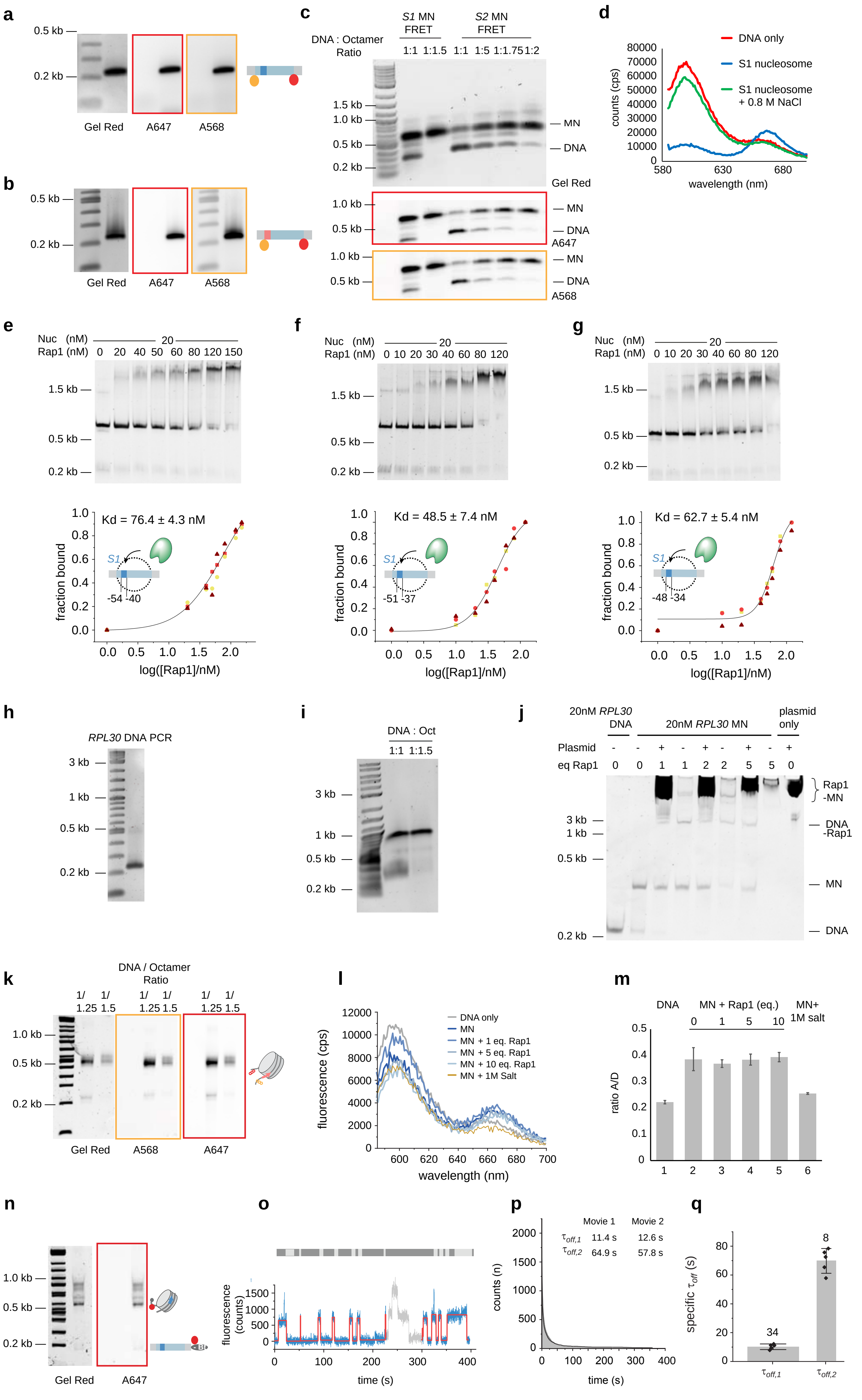

a
0.5 kb
0.2 kb
Gel Red
A647
A568
b
c
DNA : Octamer Ratio
S1 MN FRET
S2 MN FRET
1:1 1:1.5 1:1 1:5 1:1.75 1:2
1.5 kb
1.0 kb
MN
DNA
Gel Red
d
DNA only
S1 nucleosome
S1 nucleosome + 0.8 M NaCl
counts (cps)
wavelength (nm)
e
f
g
Nuc (nM)
Rap1 (nM)
20
fraction bound
log([Rap1]/nM)
Kd = 76.4 ± 4.3 nM
Kd = 48.5 ± 7.4 nM
Kd = 62.7 ± 5.4 nM
-54 -40
-51 -37
-48 -34
h
RPL30 DNA PCR
3 kb
1 kb
i
DNA : Oct
1:1 1:1.5
j
20nM RPL30 DNA
20nM RPL30 MN
plasmid only
Plasmid
eq Rap1
Rap1 -MN
DNA -Rap1
k
DNA / Octamer Ratio
1/ 1.25 1/ 1.5
l
DNA only
MN
MN + 1 eq. Rap1
MN + 5 eq. Rap1
MN + 10 eq. Rap1
MN + 1M Salt
fluorescence (cps)
m
DNA
MN + Rap1 (eq.)
MN+ 1M salt
ratio A/D
n
o
fluorescence (counts)
time (s)
p
Movie 1
Movie 2
τoff,1 11.4 s 12.6 s
τoff,2 64.9 s 57.8 s
counts (n)
q
specific τoff (s)
34
8

FIGURE S5

Figure S5. Related to Figure 4. | Ensemble FRET nucleosome assembly:

a) PCR generation of dual labelled 1x601 P3 pieces containing Rap1 *S1* binding site (**MN_*S1*_FRET**).

b) PCR generation of dual labelled 1x601 P3 pieces containing Rap1 *S2* binding site (**MN_*S2*_FRET**).

c) Gel showing nucleosome formation by titration of refolded octamers and dialysis. Both *S1* and *S2* nucleosome assembly are visible *S1* shows complete saturation, whereas *S2* nucleosomes contain traces of free DNA.

d) FRET experiment with free DNA (**MN_*S1*_FRET**), the corresponding nucleosome, and nucleosomes incubated with 0.8 M NaCl.

e) Rap1 binding to MNs containing **P3_*S1***.

f) Rap1 binding to MNs containing **P3_*S1+3***.

g) Rap1 binding to MNs containing **P3_*S1+6***.

h) Large-scale PCR of labelled **P3_*RPL30*** DNA.

i) Refolded octamers are titrated into purified **P3_*RPL30*** DNA and undergo dialysis from 2 M to 0.1 M KCl. These are then run on 0.8% agarose gel in 0.25 TB. Saturation of nucleosomes occurs at DNA to Octamer ratio of 1:1.5.

j) To assay *RPL30* MN stability, Rap1 was titrated to 1, 2 and 5 equivalents of *RPL30* MN and left at room temperature for 10 min. To recover the nucleosomes (that were bound by Rap1), buffer DNA was added. Nucleosome integrity is ensured as no sub-nucleosomal bands (below the nucleosome band, MN) are observed.

k) Gel showing nucleosome formation by titration of refolded octamers and dialysis. MNs made using 1:1.25 DNA/octamer ratio were used for ensemble FRET experiments.

l) Emission spectra shown for **MN_*Rpl30_S1*_FRET** nucleosomes only, **MN_*Rpl30_S1*_FRET** DNA only, **MN_*Rpl30_S1*_FRET** nucleosomes with 1 equivalent (eq), 5 eq and 10 eq of Rap1-Halo. Additionally, **MN_*Rpl30_S1*_FRET** nucleosomes were incubated in 1 M NaCl.

m) Acceptor : donor (A/D) fluorescence emission ratios are plotted for two independent experiments, demonstrating that Rap1 addition does not distort nucleosome structure.

n) 5% polyacrylamide gel of RPL30 MNs (**MN_*Rpl30_S1***) for TIRF assays. The diffuse band suggests multiple nucleosome conformations.

o) Kinetic trace of Rap1-nucleosome interactions using *RPL30* MNs.

p) Cumulative histograms of 2 different movies taken in the same channel with a time interval of 30 min between acquisition. These two plots superimpose, suggesting that the incubation of MNs with Rap1 does not alter nucleosome position.

q) Bar chart of specific τ_{off}. For numerical values see **Table S4**.

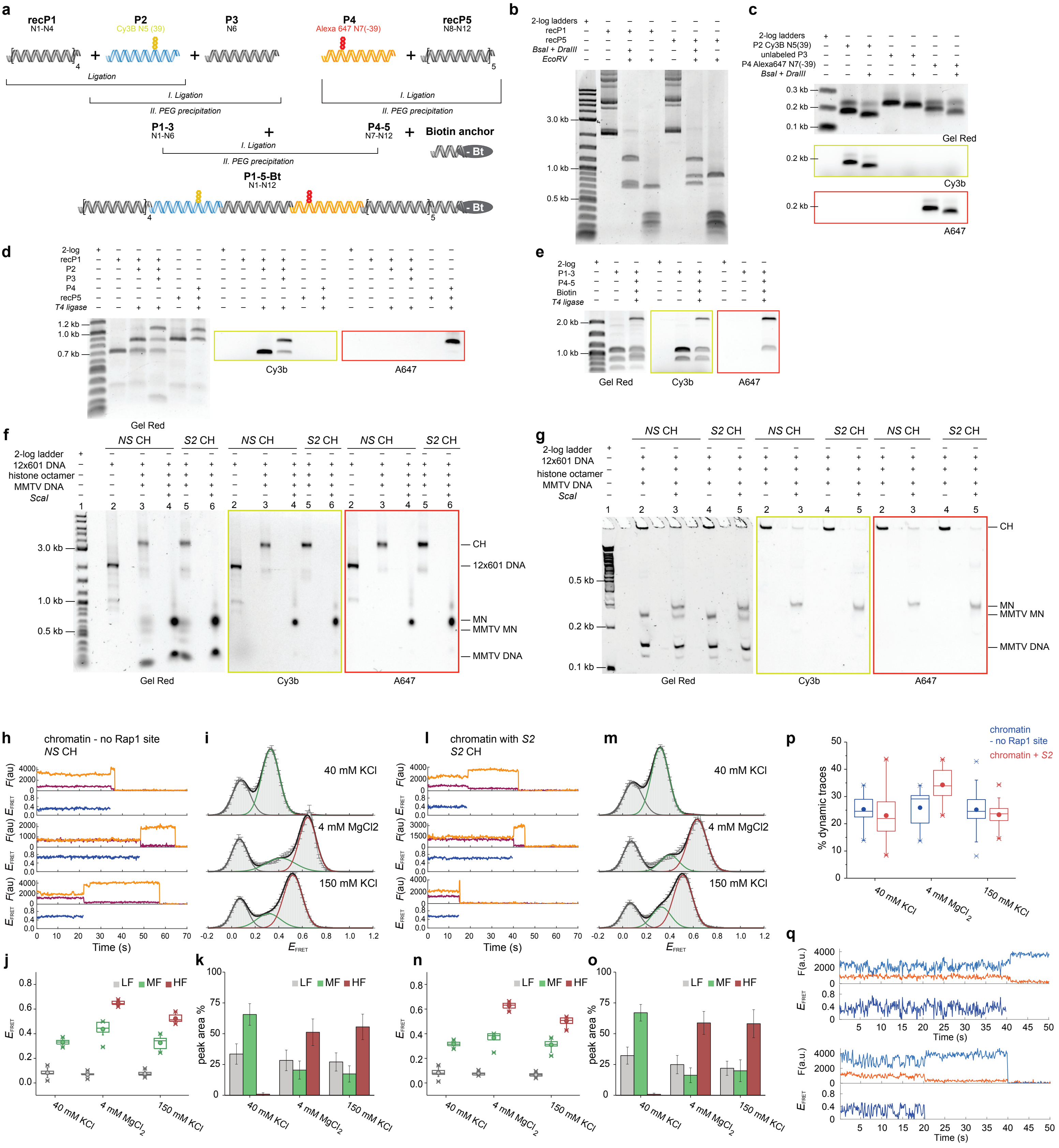

a
recP1
N1-N4
P2
Cy3B N5 (39)
P3
N6
P4
Alexa 647 N7(-39)
recP5
N8-N12
Ligation
I. Ligation
II. PEG precipitation
P1-3
N1-N6
P4-5
N7-N12
Biotin anchor
Bt
P1-5-Bt
N1-N12
b
2-log ladders
recP1
recP5
BsaI + DraIII
EcoRV
3.0 kb
1.0 kb
0.5 kb
c
2-log ladders
P2 Cy3B N5(39)
unlabeled P3
P4 Alexa647 N7(-39)
BsaI + DraIII
0.3 kb
0.2 kb
0.1 kb
Gel Red
Cy3b
A647
d
2-log
recP1
P2
P3
P4
recP5
T4 ligase
1.2 kb
1.0 kb
0.7 kb
Gel Red
Cy3b
A647
e
2-log
P1-3
P4-5
Biotin
T4 ligase
2.0 kb
1.0 kb
Gel Red
Cy3b
A647
f
NS CH
S2 CH
2-log ladder
12x601 DNA
histone octamer
MMTV DNA
ScaI
3.0 kb
1.0 kb
0.5 kb
CH
12x601 DNA
MN
MMTV MN
MMTV DNA
Gel Red
Cy3b
A647
g
NS CH
S2 CH
2-log ladder
12x601 DNA
histone octamer
MMTV DNA
ScaI
0.5 kb
0.2 kb
0.1 kb
CH
MN
MMTV MN
MMTV DNA
Gel Red
Cy3b
A647
h
chromatin - no Rap1 site
NS CH
F(au)
E_FRET
Time (s)
i
40 mM KCl
4 mM MgCl2
150 mM KCl
E_FRET
l
chromatin with S2
S2 CH
F(au)
E_FRET
Time (s)
m
40 mM KCl
4 mM MgCl2
150 mM KCl
E_FRET
p
chromatin
- no Rap1 site
chromatin + S2
% dynamic traces
40 mM KCl
4 mM MgCl2
150 mM KCl
q
F(a.u.)
E_FRET
Time (s)
j
LF
MF
HF
E_FRET
40 mM KCl
4 mM MgCl2
150 mM KCl
k
peak area %
40 mM KCl
4 mM MgCl2
150 mM KCl
n
o

FIGURE S6

Figure S6. Related to Figure 5. | Synthesis of doubly-labeled 12x601 DNA:

a) Scheme of convergent assembly of doubly-labeled 12x601 DNA from 5 fragments containing 601 NPS sequences.

b) Preparation of recP1 4x601 (lane 2-4) and recP5 5x601 (lane 5-7) with non-palindromic overhangs by complete digestion first with *BsaI* and *DraIII* followed by plasmid backbone fragmentation by *EcoRV*.

c) Production of Cy3B-labeled P2 (lane 2-3), unlabeled P3 (lane 4-5) and Alexa647-labeled P4 (lane 6-7) by PCR and *BsaI/DraIII* digestion.

d) Ligation of the singly-labeled intermediate 6x601 fragments: P1-3 (lane 2-4) and P4-5 (lane 5-6).

e) Final ligation and PEG purification of the doubly-labeled biotinylated 12x601 DNA.

f,g) Agarose gel and 5% TBE polyacrylamide analysis of chromatin assembled from doubly-labeled biotinylated 12x601 DNA (**CH_*S2*_FRET** and **CH_*NS*_FRET**) and wild-type histone octamer. To avoid overloading array DNA, low-affinity MMTV buffer DNA was added. Digestion of chromatin fibers with the restriction enzyme *ScaI* liberates MNs (lanes 4&6**f**, and lanes 3&5**g**). All gels were imaged using fluorescence imaging of Cy3 (yellow frame) and Alexa647 (red frame), stained and imaged with GelRed (no border frame).

h, **l**) Individual traces of donor (green), acceptor (red) and FRET efficiency (E_{FRET}), and (**i**, **m**) histograms of E_{FRET} of a single chromatin fibers in 40 mM KCl, 4 mM $MgCl_2$ and 150 mM KCl as indicated, error bars shown as s.e.m, number of traces and parameters of Gaussian fits shown in **Table S5**.

j, **n**) Peak center and (**k**, **o**) percentage of the integral area of Gaussian fits with LF (grey, peak $E_{FRET} <$ 0.2), MF (green, $0.2 \leq E_{FRET} \leq 0.4$) and HF (red, $E_{FRET} > 0.4$). Experiments were performed on control chromatin (**CH_*NS***, **h**-**k**) in parallel with chromatin containing Rap1 site 2 (**CH_*S2***, **l**-**o**).

p) Percentage of dynamic traces seen in two types of chromatin.

q) Examples of dynamic traces, showing anticorrelated donor and acceptor fluorescence fluctuations, indicating conformational dynamics. Data are shown for chromatin fibers with *S2* (**CH_*S2***) in the presence of 200 pM Rap1.

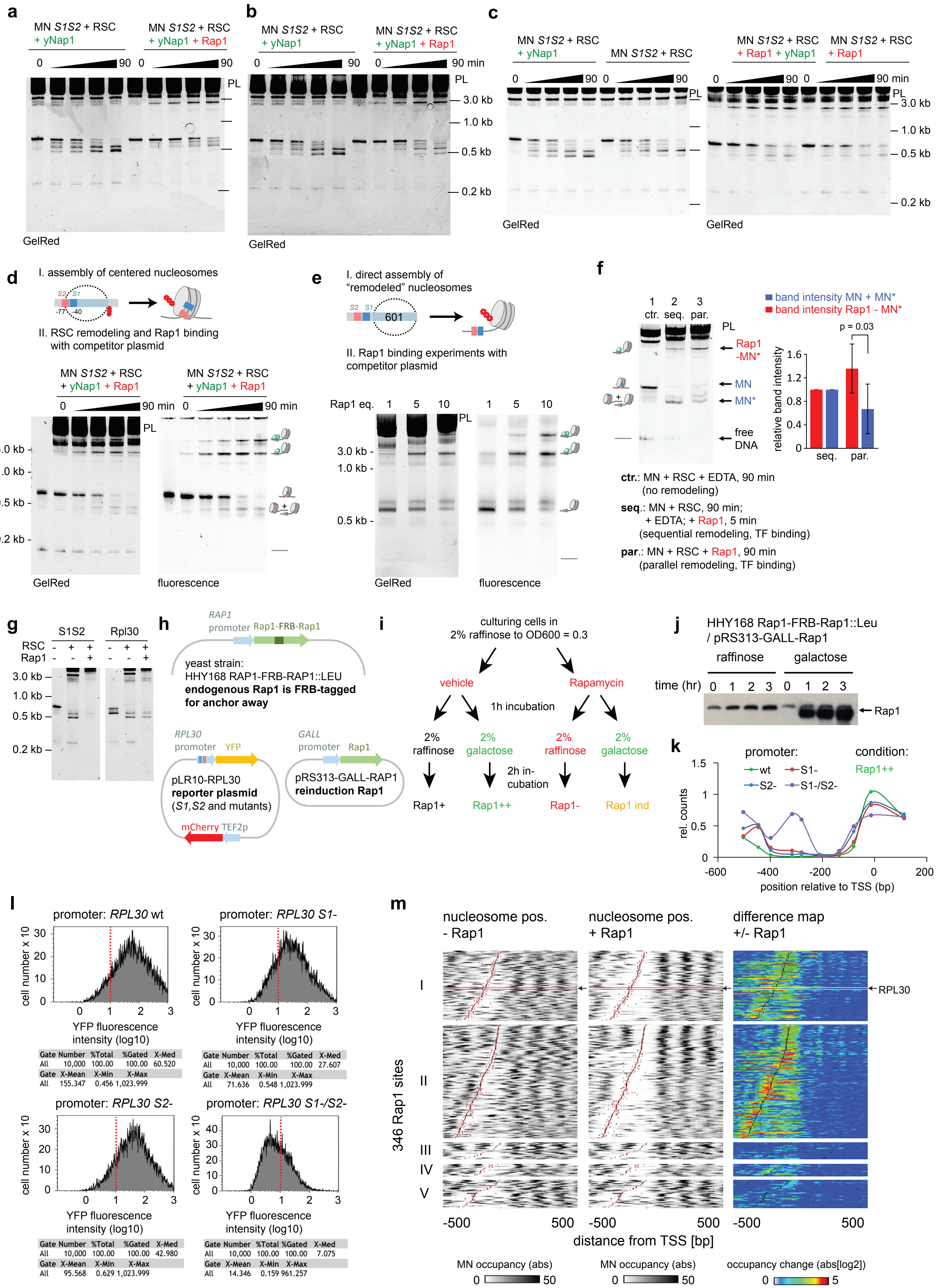
a
MN S1S2 + RSC + yNap1
MN S1S2 + RSC + yNap1 + Rap1
0 90 0 90
PL
GelRed
b
MN S1S2 + RSC + yNap1
MN S1S2 + RSC + yNap1 + Rap1
0 90 0 90 min
PL
3.0 kb
1.0 kb
0.5 kb
0.2 kb
GelRed
c
MN S1S2 + RSC + yNap1
MN S1S2 + RSC
MN S1S2 + RSC + Rap1 + yNap1
MN S1S2 + RSC + Rap1
0 90 0 90 0 90 0 90 min
PL
3.0 kb
1.0 kb
0.5 kb
0.2 kb
GelRed
d
I. assembly of centered nucleosomes
S2 S1
-77 -40
II. RSC remodeling and Rap1 binding with competitor plasmid
MN S1S2 + RSC + yNap1 + Rap1
MN S1S2 + RSC + yNap1 + Rap1
0 90 min 0 90 min
PL
3.0 kb
1.0 kb
0.5 kb
0.2 kb
GelRed
fluorescence
e
I. direct assembly of "remodeled" nucleosomes
S2 S1 601
II. Rap1 binding experiments with competitor plasmid
Rap1 eq. 1 5 10 1 5 10
PL
3.0 kb
1.0 kb
0.5 kb
GelRed
fluorescence
f
1 ctr. 2 seq. 3 par.
PL
Rap1 -MN*
MN
MN*
free DNA
band intensity MN + MN*
band intensity Rap1 - MN*
p = 0.03
relative band intensity
1.6 1.2 0.8 0.4 0
seq. par.
ctr.: MN + RSC + EDTA, 90 min (no remodeling)
seq.: MN + RSC, 90 min; + EDTA; + Rap1, 5 min (sequential remodeling, TF binding)
par.: MN + RSC + Rap1, 90 min (parallel remodeling, TF binding)
g
S1S2 Rpl30
RSC - + + - + +
Rap1 - - + - - +
3.0 kb
1.0 kb
0.5 kb
0.2 kb
h
RAP1 promoter Rap1-FRB-Rap1
yeast strain: HHY168 RAP1-FRB-RAP1::LEU
endogenous Rap1 is FRB-tagged for anchor away
RPL30 promoter YFP
pLR10-RPL30 reporter plasmid (S1,S2 and mutants)
mCherry TEF2p
GALL promoter Rap1
pRS313-GALL-RAP1 reinduction Rap1
i
culturing cells in 2% raffinose to OD600 = 0.3
vehicle
Rapamycin
1h incubation
2% raffinose
2% galactose
2% raffinose
2% galactose
2h in-cubation
Rap1+
Rap1++
Rap1-
Rap1 ind
j
HHY168 Rap1-FRB-Rap1::Leu / pRS313-GALL-Rap1
raffinose galactose
time (hr) 0 1 2 3 0 1 2 3
Rap1
k
promoter:
wt S1-
S2- S1-/S2-
condition:
Rap1++
rel. counts
1.5 1 0.5 0
-600 -400 -200 0 200
position relative to TSS (bp)
l
promoter: RPL30 wt
cell number x 10
YFP fluorescence intensity (log10)
Gate Number %Total %Gated X-Med
All 10,000 100.00 100.00 60.520
Gate X-Mean X-Min X-Max
All 155.347 0.456 1,023.999
promoter: RPL30 S1-
Gate Number %Total %Gated X-Med
All 10,000 100.00 100.00 27.607
Gate X-Mean X-Min X-Max
All 71.636 0.548 1,023.999
promoter: RPL30 S2-
Gate Number %Total %Gated X-Med
All 10,000 100.00 100.00 42.980
Gate X-Mean X-Min X-Max
All 95.568 0.629 1,023.999
promoter: RPL30 S1-/S2-
Gate Number %Total %Gated X-Med
All 10,000 100.00 100.00 7.075
Gate X-Mean X-Min X-Max
All 14.346 0.159 961.257
m
nucleosome pos. - Rap1
nucleosome pos. + Rap1
difference map +/- Rap1
RPL30
346 Rap1 sites
I II III IV V
-500 500 -500 500 -500 500
distance from TSS [bp]
MN occupancy (abs) 0 50
MN occupancy (abs) 0 50
occupancy change (abs[log2]) 0 5

FIGURE S7

Figure S7. Related to Figure 6| RSC remodeling in the presence of Rap1.

a,b) Replicates of gels to show RSC MN remodeling in the presence and absence of Rap1. PL is the competitor plasmid.

c) RSC remodeling of nucleosomes (**MN_S1S2**) in the presence and absence of 10 eq. yNap1 and in the absence (top) or presence (bottom) of Rap1.

d) RSC remodeling and Rap1 binding assay using nucleosomes containing TMR labelled H2A (at position 110, (Fierz et al., 2011)).

e) Rap1 binding assay using nucleosomes, reconstituted using DNA template P3_*S1S2_remodelled* (**Table S1**) with a nucleosome downstream from the Rap1 sites. These nucleosomes (which contain a fluorescent label in H2A) model the final product of RSC remodeling. Rap1 binding results in the formation of a stable complex (which is not competed off with competitor plasmid PL), resulting in similar band shift patterns as in panel **d**).

f) Nucleosomes (Lane 1), remodeled for 90 min with RSC, stopped by EDTA addition and bound to Rap1 (Lane 2), or remodeled in the presence of RSC and Rap1 (Lane 3). MN: mono-nucleosomes, MN*: remodeled mononucleosomes, Rap1-MN*: Complex between Rap1 and remodeled mononucleosomes. Quantification was performed using densitometry of fluorescent bands. p: two-tailed Student's t-test.

g) RSC remodeling of Rap1 bound nucleosomes (**MN_S1S2** and **MN_Rpl30**) in the presence or absence of Rap1, for MNase-seq.

h) Yeast constructs: The yeast strain used expresses Rap1 tagged with FRB, which allows to anchor away Rap1 (deplete from the nucleus) upon addition of rapamycin (as anchor serves the FKBP12-tagged plasma membrane protein Pma1p). In the absence of rapamycin, Rap1 binds to the *RPL30* promoter in the reporter plasmid (potentially containing mutated sites *S1* or *S2*, for sequences see **Table S7**). Rap1 then induces the reporter gene YFP (Rap1+). Rapamycin addition results in removal of Rap1 (Rap1-). Growth of the yeast cells on galactose results in reinduction of Rap1 from a plasmid under a galactose sensitive promoter (Rap1 ind).

i) Experimental scheme: Sequential treatment with rapamycin vs. vehicle and galactose vs. raffinose results in 4 samples that are further analyzed by MNase nucleosome mapping and YFP expression by FACS.

j) Rap1 expression levels before and after induction with galactose.

k) MNase mapping of different *RPL30* promoter mutants in the Rap1++ sample (for other samples, see **Figure 6d**).

l) Determination of reporter gene induction by FACS sorting for YFP fluorescence. Promoter types wt and *S2*- show high YFP expression, for *S1*- expression is reduced and for *S1-/S2*- expression is strongly suppressed.

m) Heatmap showing absolute nucleosome occupancy change upon depletion of Rap1 (left), presence of Rap1 (center), and the difference map (right). Data is re-analyzed from (Kubik et al., 2018). Maps represent 1,000-bp-wide regions centered on the TSS and grouped according to the observed effects: I, additive effect of double depletion of RSC and Rap1; II, no effect of RSC depletion; III, no effect of Rap1 depletion, IV, enhanced effect of double depletion; V, no effect of either depletion. The regions in each group were sorted and oriented according to the distance to the nearest Rap1 site; Rap1 sites are represented by black boxes.

Table S1. Related to Figures 1-6 | Sequences of 1 x 601 pieces for recombinant and PCR-generated pieces.

Fragment	Sequence
P1	-100 -95 -90 -85 -80 -75 -70 -65 -60 -55 -50 -45 -40 -35 -30 -25<br>CACTTGGTGGCGGCCGCC**CTGGAGAATCCCGGTGCCGAGGCCGCTCAATTGGTCGTAGACAGCTCTA**<br>-20 -15 -10 -5 0 5 10 15 20 25 30 35 40 45 50 55<br>**GCACCGCTTAAACGCACGTACGCGCTGTCCCCCGCGTTTTAACCGCCAAGGGGATTACTCCCTAGTCTCCAGGCACGTGT**<br>60 65 70 75 80 85 90 95 100 105<br>**CAGATACTGCAGAGATCT**CTAGATCCATGGAGTACTTGGTCTCATAGC
P2	-100 -95 -90 -85 -80 -75 -70 -65 -60 -55 -50 -45 -40 -35 -30 -25<br>GATCGGTCTCATAGC**CTGGAGAATCCCGGTGCCGAGGCCGCTCAATTGGTCGTAGACAGCTCTA**<br>-20 -15 -10 -5 0 5 10 15 20 25 30 35 40 45 50 55<br>**GCACCGCTTAAACGCACGTACGCGCTGTCCCCCGCGTTTTAACCGCCAAGGGGATTACTCCCTAGTCTCCAGGCACGTGT**<br>60 65 70 75 80 85 90 95 100 105<br>**CAGATATATACATCCTGT**CACACTGTGGATC
P3	-100 -95 -90 -85 -80 -75 -70 -65 -60 -55 -50 -45 -40 -35 -30 -25<br>CACACTGTGCCAAGTACT**T**ACGCGGCCGCC**CTGGAGAATCCCGGTGCCGAGGCCGCTCAATTGGTCGTAGACAGCTCTA**<br>-20 -15 -10 -5 0 5 10 15 20 25 30 35 40 45 50 55<br>**GCACCGCTTAAACGCACGTACGCGCTGTCCCCCGCGTTTTAACCGCCAAGGGGATTACTCCCTAGTCTCCAGGCACGTGT**<br>60 65 70 75 80 85 90 95 100 105<br>**CAGATACTGCAGAGATCT**CTAGATCC**A**TGGAGTACTTGGTCTCAAACC
P3_*S1*	-100 -95 -90 -85 -80 -75 -70 -65 -60 -55 -50 -45 -40 -35 -30 -25<br>CACACTGTGCCAAGTACTTACGCGGCCGCCC**TGGAGAATCCCGGTGCCG**TACATCTGTGCACC**GTCGTAGACAGCTCTA**<br>-20 -15 -10 -5 0 5 10 15 20 25 30 35 40 45 50 55<br>**GCACCGCTTAAACGCACGTACGCGCTGTCCCCCGCGTTTTAACCGCCAAGGGGATTACTCCCTAGTCTCCAGGCACGTGT**<br>60 65 70 75 80 85 90 95 100 105<br>**CAGATACTGCAGAGATCT**CTAGATCC**A**TGGAGTACTTGGTCTCAAACC
P3_*S1*+3	-100 -95 -90 -85 -80 -75 -70 -65 -60 -55 -50 -45 -40 -35 -30 -25<br>CACACTGTGCCAAGTACTTACGCGGCCGCCC**TGGAGAATCCCGGTGCCGAGG**TACATCTGTGCACC**GTAGACAGCTCTA**<br>-20 -15 -10 -5 0 5 10 15 20 25 30 35 40 45 50 55<br>**GCACCGCTTAAACGCACGTACGCGCTGTCCCCCGCGTTTTAACCGCCAAGGGGATTACTCCCTAGTCTCCAGGCACGTGT**<br>60 65 70 75 80 85 90 95 100 105<br>**CAGATACTGCAGAGATCT**CTAGATCCATGGAGTACTTGGTCTCAAACC
P3_*S1*+6	-100 -95 -90 -85 -80 -75 -70 -65 -60 -55 -50 -45 -40 -35 -30 -25<br>CACACTGTGCCAAGTACTTACGCGGCCGCCC**TGGAGAATCCCGGTGCCGAGGCCG**TACATCTGTGCACC**GACAGCTCTA**<br>-20 -15 -10 -5 0 5 10 15 20 25 30 35 40 45 50 55<br>**GCACCGCTTAAACGCACGTACGCGCTGTCCCCCGCGTTTTAACCGCCAAGGGGATTACTCCCTAGTCTCCAGGCACGTGT**<br>60 65 70 75 80 85 90 95 100 105<br>**CAGATACTGCAGAGATCT**CTAGATCCATGGAGTACTTGGTCTCAAACC
P3_*S2*	-100 -95 -90 -85 -80 -75 -70 -65 -60 -55 -50 -45 -40 -35 -30 -25<br>CACACTGTGCCAAGTACTTACGCGGCACCATCCATACATT**CCGGTGCCGAGGCCGCTCAATTGGTCGTAGACAGCTCTA**<br>-20 -15 -10 -5 0 5 10 15 20 25 30 35 40 45 50 55<br>**GCACCGCTTAAACGCACGTACGCGCTGTCCCCCGCGTTTTAACCGCCAAGGGGATTACTCCCTAGTCTCCAGGCACGTGT**<br>60 65 70 75 80 85 90 95 100 105<br>**CAGATACTGCAGAGATCT**CTAGATCC**A**TGGAGTACTTGGTCTCAAACC

P3_S2*	-100 -95 -90 -85 -80 -75 -70 -65 -60 -55 -50 -45 -40 -35 -30 -25<br>CACACTGTGCCAAGTACT**T**ACGCGGCCGCC**CTGGAGAATCCCGGTGCCG**ACCATCCATACATT**GTCGTAGACAGCTCTA**<br>-20 -15 -10 -5 0 5 10 15 20 25 30 35 40 45 50 55<br>**GCACCGCTTAAACGCACGTACGCGCTGTCCCCCGCGTTTTAACCGCCAAGGGGATTACTCCCTAGTCTCCAGGCACGTGT**<br>60 65 70 75 80 85 90 95 100 105<br>**CAGATACTGCAGAGATCT**CTAGATCC**A**TGGAGTACTTGGTCTCAAACC
P3_S1S2	-100 -95 -90 -85 -80 -75 -70 -65 -60 -55 -50 -45 -40 -35 -30 -25<br>CACACTGTGCCAAGTACT**T**ACGCGGCACCATCCATACATT**CCGGTGCCG**TACATCTGTGCACC**GTCGTAGACAGCTCTA**<br>-20 -15 -10 -5 0 5 10 15 20 25 30 35 40 45 50 55<br>**GCACCGCTTAAACGCACGTACGCGCTGTCCCCCGCGTTTTAACCGCCAAGGGGATTACTCCCTAGTCTCCAGGCACGTGT**<br>60 65 70 75 80 85 90 95 100 105<br>**CAGATACTGCAGAGATCT**CTAGATCC**A**TGGAGTACTTGGTCTCAAACC
P3_S1S2_remodelled	-125 -120 -115 -110 -105 -100 -95 -90 -85 -80 -75 -70 -65 -60 -55 -50<br>TACTTACGCGGCACCATCCATACATTCCGGTGCCGTACATCTGTGCACCGTAGC**CTTGGAGAATCCGGTGCCGAGGCC**<br>-45 -40 **-35** **-30** **-25** **-20** **-15** 10 -5 0 5 10 15 20 25 30<br>**GCTCAATTGGTCGTAGACAGCTCTAGCACCGCTTAAACGCACGTACGCGCTGTCCCCCGCGTTTTAACCGCCAAGGGGAT**<br>35 40 45 50 55 60 65 70 75 80<br>**TACTCCCTAGTCTCCAGGCACGTGTCAGATATATACATCCTGT**CACACT
P3_RPL30	-100 -95 -90 -85 -80 -75 -70 -65 -60 -55 -50 -45 -40 -35 -30 -25<br>CACACTGTGCCAAGTACT**AAGACCTT**ACCATCCATACATT**TTGGAACGT**TACATCTGTGCACC**ACATATTTTTGATCTC**<br>-20 -15 -10 -5 0 5 10 15 20 25 30 35 40 45 50 55<br>**AGATTTTAGTGTTTTTTTTTTTGGTCCTTGTTGAACCTCTTATTTCCCGCCTACAAAGTAATGATCCTTACTGCGGTGCT**<br>60 65 70 75 80 85 90 95 100 105<br>**AGATGGGGGTTCAGTCTCTCCAGGCAGGAC**AGTACTTGGTCTCAAACC
P3_RPL30_S1	-100 -95 -90 -85 -80 -75 -70 -65 -60 -55 -50 -45 -40 -35 -30 -25<br>CACACTGTGCCAAGTAC**TAAGACCTTACCAGTCAGACATTTTGGAACGT**TACATCTGTGCACC**ACATATTTTTGATCTC**<br>-20 -15 -10 -5 0 5 10 15 20 25 30 35 40 45 50 55<br>**AGATTTTAGTGTTTTTTTTTTTGGTCCTTGTTGAACCTCTTATTTCCCGCCTACAAAGTAATGATCCTTACTGCGGTGCT**<br>60 65 70 75 80 85 90 95 100 105<br>**AGATGGGGGTTCAGTCTCTCCAGGCAAGAC**AGTACTTGGTCTCAAACC
P4	-100 -95 -90 -85 -80 -75 -70 -65 -60 -55 -50 -45 -40 -35 -30 -25<br>GATCGGTCTCAAACC**CTGGAGAATCCCGGTGCCGAGGCCGCTCAATTGGTCGTAGACAGCTCTA**<br>-20 -15 -10 -5 0 5 10 15 20 25 30 35 40 45 50 55<br>**GCACCGCTTAAACGCACGTACGCGCTGTCCCCCGCGTTTTAACCGCCAAGGGGATTACTCCCTAGTCTCCAGGCACGTGT**<br>60 65 70 75 80 85 90 95 100 105<br>**CAGATATATACATCCTGT**CACGTCGTGGATC
P5	-100 -95 -90 -85 -80 -75 -70 -65 -60 -55 -50 -45 -40 -35 -30 -25<br>CACGTCGTGCCAAGTACTTACGCGGCCGCC**CTGGAGAATCCCGGTGCCGAGGCCGCTCAATTGGTCGTAGACAGCTCTA**<br>-20 -15 -10 -5 0 5 10 15 20 25 30 35 40 45 50 55<br>**GCACCGCTTAAACGCACGTACGCGCTGTCCCCCGCGTTTTAACCGCCAAGGGGATTACTCCCTAGTCTCCAGGCACGTG**<br>60 65 70 75 80 85 90 95 100 105<br>**TCAGATACTGCAGAGATCT**CTAGATCCGGTCTCACTAA

601 or native RPL30 sequences indicated in bold. The labeled base pairs are indicated in red. The numbering is given as number of base-pairs relative to the dyad in the 601 sequence. Rap1 binding sites are indicated in blue for S1 and burgundy for S2.

Table S2. Related to Figures 1-6 | Sequences of all labeled oligonucleotides

Description	Dye	Sequence
P2_pos39_rev	Cy3B	5'- GATCCACAGTGTGACAGGATGTATATATCTGA CACGTGCCTGGAGACTAGGGAG-3'
P3_pos82_rev	Alexa 647	5'-GATCGCGGTTTGAGACCAAGTACTCCA/iAmMC6T/ GGATCTAGAGATCTCTGC-3'
P3_pos-86_fwd	Alexa 568	5'-GATCGCACACTGTGCCAAGTACT/iAmMC6T/AC GCGGCCGCCCTGGAGAATCC-3'
P3_pos-86_*S2*_fwd	Alexa 568	5'-GATCGCACACTGTGCCAAGTACT/iAmMC6T/AC GCGGCACCATCCATACATTCC-3'
RPL30_S1_pos-86_fwd	Alexa 568	5'-GATCGCACACTGTGCCAAGTACT/iAmMC6T/AGA CCTTACCAGTCAGACATTTTGG-3'
RPL30_S1_pos82_rev	Alexa 647	5'-GATCGCGGTTTGAGACCAAGTACTGTC/iAmMC6T/ TGCCTGGAGAGACTGAACCC-3'
P4_pos-39_rev	Alexa 647	5'-GATCGGTCTCAAACCCTGGAGAATCCCGGTGCCG AGGCCGCTCAATTGGTCGTAGACAGC-3'
P3_Anchor_fwd	-	5'-ph-AACCTAGTCTGCTCAGTACTCGTCGCTAGATCC ATGGTCCGATTACGCGG-3'
P3_RPL30_Anchor_fwd	Alexa 647	5'-ph-AACCTAGTCTGCTCAGTAC/iAmMC6T/CGTCG CTAGATCCATGGTCCGATTACGCGG-3'
P5_Anchor_fwd	-	5'-ph-CTAATAGTCTGCTCAGTACTCGTCGCTAGATC CATGGTCCGATTACGCGG-3'
Anchor_rev	-	5'-biotin-CCGCGTAATCGGACCATGGATCTAGCG ACGAGTACTGAGCAGACTA-3'

Table S3. Related to Figures 1-6 | Overview of all chromatin DNA with different combinations of labels.

Experiment	Name	Backbone	Dye	Modification
Rap1 nucleosome binding	MN *S1*<br>MN *S2*<br>MN *S2**<br>MN *S1S2*<br>MN RPL30 S1<br>MN *NS*	P3_*S1*<br>P3_*S2*<br>P3_*S2**<br>P3_*S1S2*<br>P3_RPL30_S1<br>P3	Alexa647 (82)<br>Alexa647 (82)<br>Alexa647 (82)<br>Alexa647 (82)<br>Alexa647 (P3_RPL30_Anchor)<br>Alexa647 (82)	3' biotin<br>3' biotin<br>3' biotin<br>3' biotin<br>3' biotin<br>3‘ biotin
Rap1 chromatin binding	CH *S1*<br>CH *S2*<br>CH *NS*	P1P2 ; P3_*S1* ; P4P5<br>P1P2 ; P3_*S2* ; P4P5<br>P1P2 ; P3 ; P4P5	Alexa647 (82)<br>Alexa647 (82)<br>Alexa647 (82)	3' biotin<br>3' biotin<br>3' biotin
Nucleosome FRET	MN *S1* FRET<br>MN *S2* FRET<br>MN RPL30 S1 FRET	P3_*S1*<br>P3_*S2*<br>P3_RPL30_S1	P3: Alexa647 (82), Alexa568 (-86)<br>P3: Alexa647 (82), Alexa568 (-86)<br>RPL30: Alexa647 (82), Alexa568 (-86)	
Chromatin FRET	CH *NS* FRET<br>CH *S2* FRET	P1 ; P2 ; P3 ; P4 ; P5<br>P1 ; P2 ; P3_*S2* ; P4 ; P5	P2: Cy3B (39), P4: Alexa647 (-39)<br>P2: Cy3B (39), P4: Alexa647 (-39)	3' biotin<br>3' biotin
Ensemble binding & RSC experiments	MN *RPL30*<br>MN *S1S2*	P3_*RPL30*<br>P3_*S1S2*		

Table S4. Related to Figure 1-3 | All kinetic parameters of Rap1 interacting with DNA, nucleosomes and chromatin.

	dissociation kinetics						binding kinetics		statistics
	dwell time (s)			Amplitude (%)			rate constants (x 10^7 s-1)		
	$\tau_{off,0}$	$\tau_{off,1}$	$\tau_{off,2}$	A_0	A_1	A_2	k_{on}	$k_{on,specific}$	n exp.
**DNA**									
DNA *S2*	X	12.4 ± 4.5	451.5 ± 115*	X	35 ± 17	65 ± 17	6.3 ± 1.9	6.3 ± 1.9	4
**Nucleosomes**									
MN S1	0.6 ± 0.4	17.8 ± 10.8	116.4 ± 36.0‡	43 ± 5	27 ± 9	30 ± 7	7.2 ± 2.9	4.0 ± 2	4
MN S2	0.7 ± 0.2	8.4 ± 1.4	46.1 ± 3.0†	35 ± 6	54 ± 4.9	11 ± 4	11 ± 2.7	7.1 ± 2	4
MN S2*	0.3 ± 0.1	2.4 ± 0.4	7.7 ± 1.9	17 ± 5	51 ± 10	32 ± 8	18 ± 5	15 ± 5	5
MN no site	0.2 ± 0.1	3.5 ± 3.0	X	83 ± 10	17 ± 10	X	4.1 ± 3.4	0.5 ± 0.4	4
MN RPL30_S1	1.3 ± 0.2	10.4 ± 1.7	69.6 ± 8.3	58 ± 4	34 ± 5	8 ± 7	27 ± 5	11 ± 2	5
**Chromatin**									
CH *S1*	0.6 ± 0.1	3.2 ± 0.6	25.6 ± 4.0	73 ± 12.9	24 ± 11	4 ± 2	1.00E+07	7.1 ± 2	8
CH *S2*	0.6 ± 0.2	2.6 ± 0.6	16.8 ± 2.9	69 ± 10	27 ± 9	4 ± 2	33 ± 17	9.2 ± 6.6	6
CH no site	0.2 ± 0.1	1.4 ± 0.7	X	81 ± 2	18.7 ± 2	X	17 ± 4	3.0 ± 1.1	3

Reported are values uncorrected for photobleaching. For photobleaching correction, see **Star Methods**. For the longest time constants, corrected values are reported below.

*: The bleaching time of JF-549 is 168 s (Figure S4). Due to stroboscopic imaging, the DNA sample is only illuminated 14% of the time, extending the photon budget. If the experimental value is corrected for photobleaching, $\tau_{off,2}$ = 721 ± 183 s.

‡: If the experimental value is corrected for photobleaching, $\tau_{off,2}$ = 330 ± 102 s.

†: If the experimental value is corrected for photobleaching, $\tau_{off,2}$ = 63 ± 4.3 s.

Table S5. Related to Fig. 5 | Summary of Gaussian fits and percentage of dynamic traces from chromatin compaction experiments.

		CH *NS* FRET			CH *S2* FRET		
		40 mM KCl	4 mM Mg^{2+}	150 mM KCl	40 mM KCl	4 mM Mg^{2+}	150 mM KCl
**LF**	A_1	0.04 ± 0.01	0.03 ± 0.01	0.03 ± 0.01	0.04 ± 0.01	0.03 ± 0.01	0.03 ± 0.01
	c_1	0.08 ± 0.03	0.07 ± 0.02	0.08 ± 0.02	0.08 ± 0.03	0.08 ± 0.01	0.07 ± 0.01
	σ_1	0.07 ± 0.01	0.07 ± 0.01	0.07 ± 0.01	0.07 ± 0.01	0.06 ± 0.01	0.07 ± 0.01
	% area	33.5 ± 8.3	28.3 ± 8.4	27.1 ± 7.4	32.2 ± 6.9	24.9 ± 7.4	22 ± 5.8
**MF**	A_2	0.07 ± 0.01	0.02 ± 0.01	0.02 ± 0.01	0.07 ± 0.01	0.02 ± 0.01	0.02 ± 0.01
	c_2	0.33 ± 0.02	0.44 ± 0.06	0.33 ± 0.05	0.32 ± 0.02	0.37 ± 0.05	0.31 ± 0.04
	σ_2	0.07 ± 0.01	0.07 ± 0.01	0.08 ± 0.01	0.07 ± 0.01	0.08 ± 0.01	0.07 ± 0.01
	% area	65.6 ± 8.8	20.5 ± 7.3	17.4 ± 6.6	67 ± 6.7	16.3 ± 6	19.9 ± 8.9
**HF**	A_3	0	0.07 ± 0.02	0.06 ± 0.01	0	0.06 ± 0.02	0.06 ± 0.01
	c_3	0.55 ± 0.06	0.65 ± 0.02	0.52 ± 0.03	0.58 ± 0.13	0.63 ± 0.03	0.5 ± 0.03
	σ_3	0.03 ± 0.03	0.06 ± 0.01	0.07 ± 0.01	0.03 ± 0.02	0.08 ± 0.02	0.08 ± 0.01
	% area	0.9 ± 1.2	51.2 ± 10.8	55.6 ± 10.3	0.8 ± 1	58.8 ± 9.3	58.1 ± 11.4
**N total traces**		1189	595	1477	1409	521	1523
**N dynamic traces**		306	154	375	332	175	355
**% dynamic traces**		25.3 ± 5.6	25.9 ± 6.9	25.2 ± 7.8	23 ± 9.1	34.3 ± 6	23.3 ± 4.3
**N of repeats**		21	10	23	24	10	22

Table S6. Related to Fig. 5 | Summary of Gaussian fits and percentage of dynamic traces from chromatin remodeling induced by Rap1 invasion experiments.

		CH *NS* FRET				CH *S2* FRET			
Rap1 conc.		50 pM	100 pM	200 pM	500 pM	50 pM	100 pM	200 pM	500 pM
LF	A_1	0.03 ± 0.01	0.02 ± 0.01	0.04 ± 0.01	0.02 ± 0.01	0.03 ± 0.01	0.03 ± 0.01	0.03 ± 0.01	0.03 ± 0.01
	c_1	0.08 ± 0.01	0.1 ± 0.02	0.08 ± 0.02	0.09 ± 0.01	0.09 ± 0.02	0.08 ± 0.02	0.08 ± 0.02	0.07 ± 0.02
	σ_1	0.07 ± 0.01	0.08 ± 0.02	0.07 ± 0.01	0.07 ± 0.01	0.07 ± 0.01	0.08 ± 0.02	0.07 ± 0.01	0.07 ± 0.01
	% area	26 ± 6.3	17.9 ± 9.7	29.2 ± 8.3	18.1 ± 7.5	28.1± 7	24.5± 6.2	26.2 ± 6.8	25.5 ± 7.8
MF	A_2	0.02 ± 0.01	0.02 ± 0	0.02 ± 0	0.01 ± 0.01	0.04 ± 0.02	0.04 ± 0.02	0.04 ± 0.02	0.05 ± 0.01
	c_2	0.33 ± 0.04	0.35 ± 0.01	0.33 ± 0.04	0.36 ± 0.03	0.37 ± 0.02	0.38 ± 0.02	0.36 ± 0.03	0.34 ± 0.03
	σ_2	0.08 ± 0.01	0.08 ± 0.01	0.08 ± 0.01	0.08 ± 0.01	0.09 ± 0.01	0.09 ± 0.01	0.1 ± 0	0.1 ± 0.01
	% area	21.2 ± 6.2	15.5 ± 4	18.6 ± 4.8	15.1 ± 5.3	43.2 ± 20.2	51.6 ± 19	53.8 ± 17.8	57.3 ± 14.2
HF	A_3	0.06 ± 0.01	0.07 ± 0.01	0.06 ± 0.01	0.07 ± 0.01	0.03 ± 0.02	0.03 ± 0.02	0.02 ± 0.02	0.02 ± 0.01
	c_3	0.53 ± 0.04	0.56 ± 0.01	0.53 ± 0.04	0.57 ± 0.01	0.56 ± 0.07	0.55 ± 0.06	0.59 ± 0.11	0.57 ± 0.06
	σ_3	0.07 ± 0.01	0.08 ± 0.01	0.07 ± 0.01	0.08 ± 0.02	0.07 ± 0.02	0.06 ± 0.02	0.08 ± 0.02	0.08 ± 0.02
	% area	52.8 ± 8.8	66.6 ± 6.3	52.3 ± 6.3	66.8 ± 7.8	28.6 ± 18	23.8 ± 17.7	20 ± 16.8	17.2 ± 9.9
N total traces		1135	169	365	212	996	927	678	477
N dynamic traces		285	36	91	38	280	284	240	146
% dynamic traces		25<br>± 6.6	21.8 ± 6.7	25.4 ± 8.7	17.7 ± 7	27.1± 7.6	31.2 ± 7	36.5 ± 10.4	31 ± 10
N of repeats		19	4	9	4	15	15	10	8

Table S7. Related to Figure 6 | *RPL30* promoter sequences in reporter plasmid for yeast experiments.

**Promoter type**	**Sequence**
RPL30_S1_S2	atatcatgcagtacattgacagtatatcacttctggataggacgccaacc<br>gccagtctgtgtggtatttcctcccgtctctgcgtgccagacgggatcag<br>cccgtctattctcgcgtcgtcctgcctggagagactqaacccccatctag<br>caccgcagtaaggatcattactttgtaggcgggaaataagaggttcaaca<br>aggaccaaaaaaaaaaacactaaaatctgagatcaaaaatatgc**ggtgca**<br>**cagatgta**acgttccaa**aatgtatggatggt**aaggtctttttccaagaaa<br>cgtatctttttttcagctagggccgcatttaaattttttttttttttcaat<br>tttttttcctattggagacggaatggcaaagctcacgctgggcgatagca<br>cgtaaccacgctggtgtattttcatcctaatcattctctttcatctattt<br>cgataagctacttaatgtcgaagttttaacttccatttgttggaatgttc<br>attacagtttatacttactccttgctcttttataatataaccaaacagac<br>cggagtgtttaagaacctacagcttattcaattaatcaatatacgcaggc
RPL30_S1mut_S2	…<br>aggaccaaaaaaaaaaacactaaaatctgagatcaaaaatatgc**ggtgAC**<br>**caACtgta**acgttccaa**aatgtatggatggt**aaggtctttttccaagaaa<br>…
RPL30_S1_S2mut	…<br>aggaccaaaaaaaaaaacactaaaatctgagatcaaaaatatgc**ggtgca**<br>**cagatgta**acgttccaa**aatgtCtgACtggt**aaggtctttttccaagaaa<br>…
RPL30_S1mut_S2mut	…<br>aggaccaaaaaaaaaaacactaaaatctgagatcaaaaatatgc**ggtgAC**<br>**caACtgta**acgttccaa**aatgtCtgACtggt**aaggtctttttccaagaaa<br>…

Rap1 binding site *S1* is indicated in blue, *S2* in burgundy. Mutated residues are given in red and highlighted in yellow.
